# Genome-wide dynamics of a bacterial response to antibiotics that target the cell envelope

**DOI:** 10.1186/1471-2164-12-226

**Published:** 2011-05-11

**Authors:** Andy Hesketh, Chris Hill, Jehan Mokhtar, Gabriela Novotna, Ngat Tran, Mervyn Bibb, Hee-Jeon Hong

**Affiliations:** 1 Department of Biochemistry, University of Cambridge, Cambridge, UK; 2 Department of Molecular Microbiology, John Innes Centre, Norwich, UK

## Abstract

**Background:**

A decline in the discovery of new antibacterial drugs, coupled with a persistent rise in the occurrence of drug-resistant bacteria, has highlighted antibiotics as a diminishing resource. The future development of new drugs with novel antibacterial activities requires a detailed understanding of adaptive responses to existing compounds. This study uses *Streptomyces coelicolor *A3(2) as a model system to determine the genome-wide transcriptional response following exposure to three antibiotics (vancomycin, moenomycin A and bacitracin) that target distinct stages of cell wall biosynthesis.

**Results:**

A generalised response to all three antibiotics was identified which involves activation of transcription of the cell envelope stress sigma factor σ^E^, together with elements of the stringent response, and of the heat, osmotic and oxidative stress regulons. Attenuation of this system by deletion of genes encoding the osmotic stress sigma factor σ^B ^or the ppGpp synthetase RelA reduced resistance to both vancomycin and bacitracin. Many antibiotic-specific transcriptional changes were identified, representing cellular processes potentially important for tolerance to each antibiotic. Sensitivity studies using mutants constructed on the basis of the transcriptome profiling confirmed a role for several such genes in antibiotic resistance, validating the usefulness of the approach.

**Conclusions:**

Antibiotic inhibition of bacterial cell wall biosynthesis induces both common and compound-specific transcriptional responses. Both can be exploited to increase antibiotic susceptibility. Regulatory networks known to govern responses to environmental and nutritional stresses are also at the core of the common antibiotic response, and likely help cells survive until any specific resistance mechanisms are fully functional.

## Background

The bacterial cell wall is a key target for antibiotic discovery; it is crucial for cell growth, and provides a physical protective barrier between the cell and its environment. Antibiotics that inhibit bacterial cell wall biosynthesis, such as penicillin and vancomycin, are extremely important in the clinical treatment of infectious diseases. Free living bacteria resident in soil and marine environments produce a vast array of chemically diverse biologically active molecules, and the actinomycete *Streptomyces *spp. in particular are a rich source of antibiotics [1,2]. Antibiotics do not kill the organisms that produce them since they have co-evolved systems that make them resistant or tolerant to their effects, but it is when similar systems develop in the target pathogenic bacteria allowing them to survive antibiotic treatments that major problems arise. The increase in the number of cases of methicillin-resistant *Staphylococcus aureus *(MRSA), and the emergence of vancomycin-resistant MRSA in hospital-acquired infections are two such examples [3-6].

Understanding how antibiotics can fail to be effective against bacteria has the potential both to provide novel insights into their mode of action, and to suggest new targets for anti-infective therapy. In this study we use *Streptomyces coelicolor *A3(2) as a model system to rigorously analyse the dynamic transcriptional response to sub-lethal concentrations of three antibiotics (vancomycin, bacitracin and moenomycin A) that target distinct stages of cell wall biosynthesis. *Streptomyces *species produce about 70% of known antibiotics and are likely to be the ultimate source of most clinically relevant antibiotic resistance genes. Consequently they possess many genes involved in sensing and responding to extracellular antibiotics, and provide an excellent opportunity to understand the processes involved. *S. coelicolor *exhibits significant resistance to vancomycin (minimum inhibitory concentration (MIC) ~80 μg/ml), bacitracin (MIC ~45 μg/ml) and moenomycin ((MIC ~300 μg/ml), and learning how the presence of these compounds, and the damage to the cell wall that they cause, is communicated to the bacterial chromosome, and how the subsequent reprogramming of gene expression acts to counteract this damage, can provide new ideas for drug discovery or for prolonging the therapeutic usefulness of existing compounds. Indeed, a biochemical analysis of the VanRS two-component sensor system of *S. coelicolor *recently defined exactly how bacteria sense vancomycin, a mechanism that triggers resistance to the only antibiotic in widespread use for the treatment of MRSA [7].

The development of techniques for studying gene transcription on a genome-wide scale allows characterisation of the response to antibiotic exposure, and several recent studies have used DNA microarrays to analyse the effect of antimicrobial compounds on bacterial strains [8-13]. However, only a minority have focused on antibiotics active against the cell wall, and these have tended to use either lethal drug concentrations, or to analyse only one time point following treatment. In this work we have studied three time points after treatment, and use the antibiotics at concentrations well below their MIC. Sub-lethal concentrations of antibiotics have been reported to be optimal for data quality in this type of study [14], perhaps because bacteriocidal levels seem ultimately to result in the same mechanism of cellular death, thereby obscuring the link between the transcriptional response to the antibiotic and its mode of action [8].

Vancomycin, a glycopeptide antibiotic, inhibits the transpeptidase reaction that occurs as one of the final steps in peptidoglycan biosynthesis not by binding to the enzyme but to the enzyme substrate, the D-alanyl-D-alanine terminus of the pentapeptide of the peptidoglycan precursor lipid II [15]. Moenomycin A is a member of the phosphoglycolipid family of antibiotics, the only natural products known to target directly the transglycosylase enzyme that catalyzes polymerization of the peptidoglycan sugar backbone prior to transpeptidase-mediated cross-linking [16]. Bacitracin is a branched cyclic dodecylpeptide antibiotic that inhibits cycling of lipid II by binding to the lipid II carrier undecaprenol pyrophosphate [17,18]. By characterising the transcriptional response over a period of 90 min following treatment with each of these antibiotics we identified expression changes common to two or more compounds, or unique to each antibiotic. These genes are likely to be involved in adaptation or resistance to the antibiotics, the former representing a generalised response to cell wall damage, and the latter the antibiotic-specific responses. Antibiotic susceptibility studies using mutant strains selected for analysis on the basis of the microarray results confirmed a role for several genes in contributing to antibiotic resistance, validating the utility of this approach.

## Results

### Exposure of *S. coelicolor *to antibiotics that inhibit different stages of peptidoglycan biosynthesis induced changes in transcription in up to 27% of the genome

**Figure 1:**
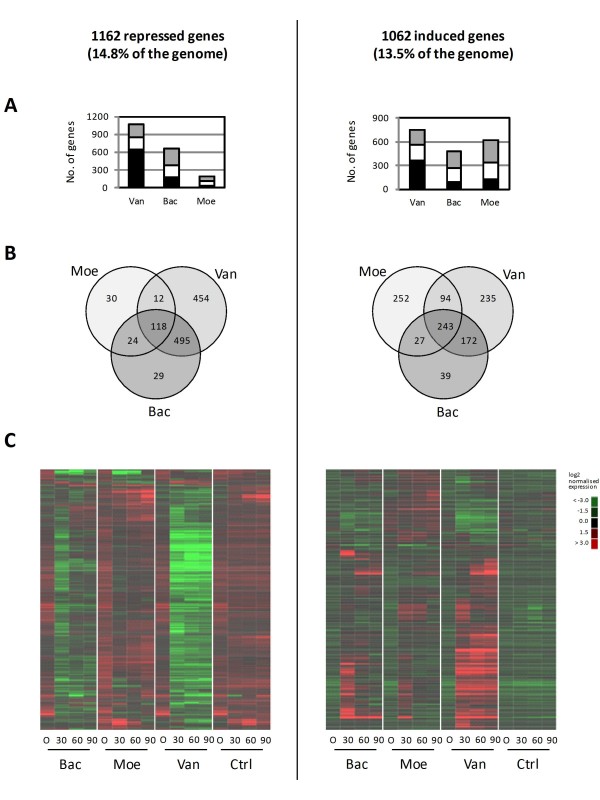
**Changes in gene expression following antibiotic treatment affect 2094 (27%) of the 7825 genes in the *S. coelicolor *genome, with many genes responding to more than one drug**. A) Genes significantly repressed (left) or induced (right) following addition of vancomycin (Van), bacitracin (Bac) or moenomycin A (Moe). Black bars represent genes that were significantly differently expressed at all three time points after treatment, white bars correspond to genes affected at any two out of the three time points, and grey bars indicate those changed at only one of the three time points. A fold-change cut-off value of 1.5 was used, and all genes are significantly differently expressed at the 1% probability level. B) Venn diagrams showing the genes from Figure 1A that respond to more than one drug, or uniquely to individual treatments. C) Heat maps showing the expression profiles of the differentially expressed genes identified in Figures 1A and 1B under all conditions analysed. The columns represent the time in minutes following the antibiotic or control (Ctrl) treatments, and the rows correspond to genes. The log2 expression values for each gene are represented by the colours according to the key scales shown, and the rows have been clustered with NeatMap using the average linkage method [88].

Transcriptome analysis identified 2094 genes whose expression was significantly different at the 1% probability level, and more than 1.5-fold up- or down-regulated, as a result of exposure of exponentially growing *S. coelicolor *cells to vancomycin, bacitracin or moenomycin A (Figure 1, Additional files 1, 2, 3 and 4). This corresponds to 27% of the 7,825 genes predicted to make up the genome [1], and includes 1162 genes that were repressed at one or more time points after antibiotic addition, and 1062 genes whose expression was up-regulated. 130 genes were common to both the repressed and induced lists. For each antibiotic treatment, the majority of the genes identified as induced or repressed were present in at least two of the three time points analysed (Figure 1A and 1C). Strikingly, only a minority (0.06%) of the 1147 genes (68/1147) changing in expression in response to bacitracin treatment responded uniquely to this compound; the vast majority were similarly affected by vancomycin or by all three drugs (see Figure 1B). In contrast, 42% (454/1079) of the genes significantly down-regulated following addition of vancomycin responded only to this compound, and 31% (235/744) were also uniquely induced. Moenomycin treatment notably down-regulated the expression of far fewer genes than either vancomycin or bacitracin, and 16% of these (30/184) responded uniquely. Moenomycin however up-regulated a similar number of genes to either vancomycin or bacitracin, approximately 60% (364/616) of which were also induced by one or more of the other antibiotics, while 40% (252/616) were uniquely induced. Interestingly, a core set of 243 genes were up-regulated in response to any one of the compounds and 118 were similarly down-regulated, representing a common response to the antibiotic treatments.

To determine the similarities and differences in the effects of each drug, the differentially expressed sets of genes illustrated in Figure 1 were subjected to gene ontology (GO) analysis (Additional files 5, 6, 7, 8, 9, 10 and 11), and compared with in-house curated sets of functionally related genes (Tables 1 and 2, Additional file 12). The latter approach was included since the computationally generated GOA annotation available for the *S. coelicolor *proteome (see Methods) has not been manually curated and is therefore incomplete. The results of these analyses are summarised in the following sections.

**Table 1:** Comparison of genes significantly induced following drug treatment with in-house curated lists of genes.

Entity list	Genes in entity list present on microarray	Vancomycin-induced (744 genes)		Bacitracin-induced (481 genes)		Moenomycin-induced (616 genes)		Induced by all (243 genes)	
					
		Genes in entity list matched with induced genes	P-Value	Genes in entity list matched with induced genes	P-Value	Genes in entity list matched with induced genes	P-Value	Genes in entity list matched with induced genes	P-Value
SigB regulon	87	39	<1E-33	33	<1E-34	42	<1E-10	29	<1E-11
Heat shock regulon	119	51	<1E-33	27	3.13E-09	43	<1E-10	26	<1E-11
ppGpp-induced genes	98	50	1.46E-25	42	<1E-34	28	1.48E-09	20	<1E-11
Gas vesicle 1	8	8	7.71E-09	7	2.81E-08	7	1.58E-07	7	2.33E-10
SigE operon	4	4	8.87E-05	4	1.54E-05	4	4.16E-05	4	9.92E-07
Ectoine biosynthesis	4	4	8.87E-05	4	1.54E-05	4	4.16E-05	4	9.92E-07
CDA	47*	39	1.96E-32	35	6.87E-33	11	0.00100	11	1.67E-07
Peptidoglycan biosynthesis (external)	16	8	4.87E-05	5	0.00234	5	0.00686	4	0.001335
Sigma factors	64	14	0.00283	14	3.23E-05	11	0.01230	8	8.76E-04
Red	23*	23	3.83E-24	9	5.25E-06				
Wbl	10	5	0.00142	3	0.02123				
SsgA like	8	4	0.00450	3	0.01089				
Cell wall hydrolyases	57			9	0.00856	14	1.23E-04		
ppGpp-repressed genes	189					28	0.00111		
Resuscitation-promoting factors	7					5	6.09E-05		
Gly Ser Thr biosynthesis	35					9	0.00140		
NRPS type II FAS	10					4	0.00589		
Amino acid metabolism	184					24	0.01225		
SigR regulon	26					6	0.01517		
Hopanoids	13					4	0.01651		
Amino acid transport	34					7	0.01675	4	0.0217
Methionine biosynthesis	9					3	0.03018		
Lysine bioysnhtesis	16					4	0.03468		
Val Leu Ile biosynthesis	25					5	0.04587		
Sensor kinase	48			10	6.56E-04				
Response regulators	94			11	0.0329				
Zur regulon	22	19	5.50E-17						
*van *cluster genes	7	7	8.00E-08						
Coelibactin	13	7	8.11E-05						

*indicates the number of probe sets rather than the number of genes.

**Table 2:** Comparison of genes significantly repressed following drug treatment with in-house curated lists of genes.

Entity list	Genes in entity list present on microarray	Vancomycin-repressed (1079 genes)		Bacitracin-repressed (666 genes)		Moenomycin-repressed (184 genes)		Repressed by all (118 genes)	
					
		Genes in entity list matched with repressed genes	P-Value	Genes in entity list matched with repressed genes	P-Value	Genes in entity list matched with repressed genes	P-Value	Genes in entity list matched with repressed genes	P-Value
Amino acid metabolism	184	69	<1E-30	49	1.21E-11	9	0.033121		
ppGpp-repressed genes	189	147	<1E-30	114	<1E-16	35	<1E-09	25	<1E-10
Ribosomal protein genes	53	44	2.09E-29	27	1.57E-15	4	0.03798	3	0.0479
tRNA synthetases	28	21	5.03E-13	18	3.77E-13	3	0.028817	3	0.0088
Conservons	51	28	8.20E-12	24	1.02E-11	11	2.37E-08	10	3.87E-09
Coelichelin	12	12	5.85E-11	12	1.72E-13	2	0.032389	2	0.014
ABC transport	227	44	0.01568	48	3.85E-09	22	2.04E-08	10	0.0025
Glutamate biosynthesis	27	8	0.02838	7	0.00694	4	0.003681	3	0.0079
LAXTG sortase substrate	14	5	0.03678	5	0.00506	4	2.68E-04	4	4.77E-05
Conservon *cvn*13	4	4	3.93E-04	4	5.96E-05	3	5.37E-05	3	1.41E-05
Conservon *cvn*5	4	4	1.53E-07	4	5.96E-05	4	3.24E-07	4	5.37E-08
ATP synthesis	8	8	8.99E-07	7	2.72E-07				
Vitamin B12 biosynthesis	11	9	1.74E-06	7	8.86E-06				
Peptidoglycan biosynthesis (cytoplasmic)	14	10	6.31E-06	9	3.65E-07				
Gly Ser Thr biosynthesis	35	16	3.93E-04	12	2.19E-05				
Conservon *cvn*10	4	4	3.93E-04	4	5.69E-05				
Conservon *cvn*12	4	4	3.93E-04	4	5.69E-05				
Conservon *cvn*1	4	4	0.00168	2	0.04030				
SigB regulon	87	23	0.00698	13	0.03697				
SigR regulon	26	9	0.01000	8	0.00120				
Conservon *cvn*4	4	3	0.00425	2	0.04030				
Methionine biosynthesis	9	5	0.00412	3	0.03702				
Phe Tyr Trp biosynthesis	33	11	0.00280	9	0.00155				
Conservon *cvn*2	3	3	2.78E-04	2	0.02138				
Val Leu Ile biosynthesis	25	11		7	0.00437				
Conservon *cvn*6	4			2	0.04030	3	5.37E-05	2	0.0013
Amino acid transport	34					5	0.001202		
Carotenoid	7					2	0.01		
Desferrioxamines	4			3	0.00245		1151		
Histidine biosynthesis	26			6	0.02161				
Lysine biosynthesis	16	8	6.83E-04						

### Common changes in expression of known stress-response genes following antibiotic treatment suggest a general environmental stress response system in *S. coelicolor*

#### i) The σ^E ^cell envelope stress response system is induced by all three drugs

**Figure 2:**
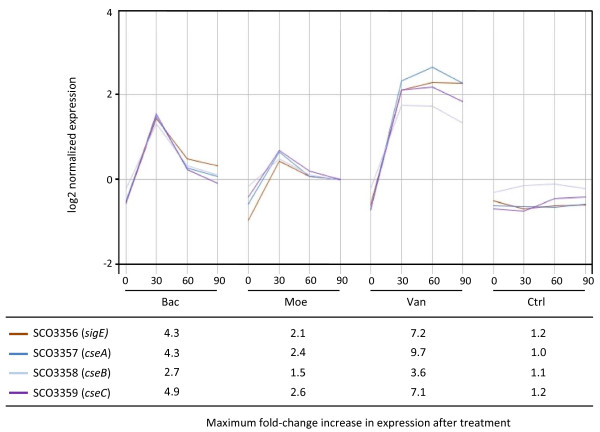
**The σ^E ^signal transduction system is induced following treatment with vancomycin, bacitracin or moenomycin**. The fold-change increase in expression at 30, 60 and 90 min following addition of vancomycin (Van), bacitracin (Bac) or moenomycin A (Moe) is shown, relative to the level of expression in the corresponding sample of the untreated control culture (Ctrl).

σ^E ^is an extracytoplasmic function sigma factor required for normal cell envelope integrity in *S. coelicolor*, and its expression is induced by a wide-variety of cell wall damaging agents [19]. Reassuringly, transcription of the four-gene operon containing the σ^E ^gene was significantly induced following treatment with each of the antibiotics (Table 1, Figure 2). In addition, similarity analysis indicated that the expression of 158 genes was closely correlated with that of σ^E ^(Pearson correlation >0.9; Additional file 13). This group is likely to include genes directly dependent on σ^E ^for their transcription, and indeed contains σ^hrdD ^(SCO3202) previously characterised to be dependent on σ^E ^for expression [20]. Interestingly, expression of the only aminoacyl-tRNA synthetase gene (SC03397) to be up-regulated in response to any drug correlates with σ^E ^transcription (similarity coefficient = 0.93); of the remaining 27 tRNA synthetase genes annotated in the genome sequence, transcription of 21 was significantly repressed following antibiotic treatment. Mutation in SCO3397, encoding a lysyl-tRNA synthetase, markedly reduced the MIC towards both vancomycin and bacitracin (Table 3) indicating that it plays an important role in resistance to these compounds.

**Table 3:** Minimum inhibitory concentration (MIC) test of mutant strains carrying disruptions in genes targeted based on their transcriptome profiles.

Strains	Relevant genotype/comments	MIC (μg/ml)			Source/reference
			
		Vancomycin	Bacitracin	Moenomycin	
*S. coelicolor *A3(2) (M600)	SCP1^- ^SCP2^-^	~80	40-45	>300	Kieser et al. 2000 [81]
ΔSCO0600 (H1001)	Δ*sigB::apr *SCP1^- ^SCP2^-^	60-70	25-30	>300	This study
ΔSCO3356 (J2130)	Δ*sigE *SCP1^- ^SCP2^-^	60-70	35-40	>300	Paget et al. 1999 [20]
ΔSCO1513 (M570)	Δ*relA *SCP1^- ^SCP2^-^	60-70	20-25	>300	Chakraburtty and Bibb. 1997 [23]
ΔSCO3089-3090 (H1003)	ΔSCO3089-3090::*apr *SCP1^- ^SCP2^-^	70-80	15-20	>300	This study
ΔSCO3110-3111 (H1002)	ΔSCO3110-3111::*apr *SCP1^- ^SCP2^-^	70-80	30-35	>300	This study
ΔSCO3089-3090 + ΔSCO3110-3111 (H1004)	ΔSCO3089-3090 + 3110-3111::*apr *SCP1^- ^SCP2^-^	70-80	<10	>300	This study
SCO4005::Tn (M900)	SCO4005::Tn SCP1^- ^SCP2^-^	70-80	25-30	>300	Hesketh et al. 2007 [21]
SCO5147::Tn (M903)	SCO5147::Tn SCP1^- ^SCP2^-^	80-90	40-45	>300	Hesketh et al. 2007 [21]
ΔSCO1875 (H1005)	ΔSCO1875::*apr *SCP1^- ^SCP2^-^	<50	10-15	>300	This study
ΔSCO2608 (H1006)	ΔSCO2608::*apr *SCP1^- ^SCP2^-^	60-70	40-45	>300	This study
SCO2897::Tn (H1007)	ΔSCO2897::Tn SCP1^- ^SCP2^-^	~60	30-35	>300	This study
SCO3580::Tn (H1008)	ΔSCO3580::Tn SCP1^- ^SCP2^-^	<50	40-45	>300	This study
SCO3771::Tn (H1009)	ΔSCO3771::Tn SCP1^- ^SCP2^-^	<50	40-45	>300	This study
ΔSCO3847 (H1010)	ΔSCO3847::*apr *SCP1^- ^SCP2^-^	~60	40-45	>300	This study
SCO3901::Tn (H1011)	ΔSCO3901::Tn SCP1^- ^SCP2^-^	~60	40-45	>300	This study
ΔSCO4013 (H1012)	ΔSCO4013::*apr *SCP1^- ^SCP2^-^	70-80	40-45	>300	This study
SCO5039::Tn (H1013)	ΔSCO5039::Tn SCP1^- ^SCP2^-^	60-70	40-45	>300	This study
ΔSCO3397 (J3355)	ΔSCO3397::*apr *SCP1^- ^SCP2^-^	<50	10-15	>300	This study

#### ii) Effects caused by exposure to cell wall antibiotics show significant similarity to those resulting from synthesis of the stringent factor ppGpp

The stringent factor ppGpp is an intracellular signalling molecule that is synthesised in *S. coelicolor *in response to amino acid limitation, and which co-ordinates an adaptive response to the changing nutritional conditions that includes down-regulation of processes associated with active growth, and up-regulation of systems that may help it survive the nutritional stress [21]. Comparison of the lists of genes that were down-regulated in response to antibiotic treatment with the genes shown to be repressed following controlled induction of ppGpp synthesis [21] revealed a significant commonality for each of the compounds (Table 2). The 118 genes down-regulated in response to all three antibiotics were also significantly enriched for ppGpp-repressed genes, containing 25/189 (p-value < 1E-10; Additional file 12). These included 9 genes from conservons 5, 6 and 13. Conservons are conserved operons believed to encode membrane-associated signalling complexes [22], and more than half of the 51 *cvn *genes (from *cvns *1, 2, 4, 5, 6, 10, 12 and 13) were repressed by bacitracin or vancomycin, while 11 (from *cvns *5, 6, and 13) were repressed by moenomycin. In addition, ppGpp-induced genes were significantly over-represented in each of the vancomycin-, bacitracin-, and moenomycin-induced lists (Table 1), and in the 243 genes commonly induced by all of the antibiotics (Additional file 12).

To investigate the possibility that an increase in ppGpp synthesis helps mediate the observed responses to the antibiotic treatments, intracellular nucleotide pools were measured in cultures following exposure to vancomycin. No increase in ppGpp synthesis was observed at 15, 30 or 60 min following addition of vancomycin, but ATP and GTP pools were reduced on average by 23% and 35% after 15 min, respectively (data not shown). MIC tests indicated that a *relA *mutant strain that is incapable of ppGpp synthesis [23] is more susceptible to both bacitracin and vancomycin (Table 3).

#### iii) Antibiotic treatment induces changes in expression of many genes from the heat, osmotic and oxidative stress regulons

Recent studies by Bucca et al. [24] and Lee et al. [25] characterised the heat-shock regulon and σ^B^-dependent salt-stress regulon in *S. ceolicolor*, identifying 119 and 87 genes, respectively. Comparison of the genes significantly differently expressed in response to antibiotic treatment in this study indicated a significant overlap between the sets of data (Tables 1 and 2). The 744 genes up-regulated in response to vancomycin contained 51 of the 119 genes reported by [24] to be up-regulated in response to heat shock, an over-representation with a significance p-value of <1 E-33. Almost half of the σ^B^-dependent salt-stress regulon genes (39/87) were similarly present (p-value <1 E-33), while the 616 and 481 genes induced by moenomycin and bacitracin, respectively, contained 42/87 and 33/87 of the salt stress genes, and 43/119 and 27/119 of the heat shock genes, respectively. Indeed, of the 243 genes commonly up-regulated in response to all three antibiotics, 26 belonged to the heat shock regulon and 29 to the σ^B^-dependent salt-stress regulon. A σ^B^-deletion mutant showed a lowered MIC for both bacitracin and vancomycin when compared to the parent strain (Table 3), indicating an important role for σ^B ^in responding to cell envelope stress.

Genes from the well-characterised σ^R ^regulon responsible for disulphide bond homeostasis in response to oxidative stress [26] were significantly over-represented in both the vancomycin- and bacitracin-repressed gene lists, suggesting a decrease in σ^R^-mediated transcriptional activity after antibiotic treatment.

#### iv) Vancomycin, bacitracin and moenomycin up-regulate ectoine biosynthesis and genes for gas vesicle production

Ectoine is a pyrimidine carboxylic acid derivative that serves as a compatible solute in several bacterial species, protecting against salt and also temperature stress [27-32]. Exogenous addition of ectoine and 5-hydroxyectoine to *S. coelicolor *cultures confers protection against salt and heat stress, and a cluster of genes (SCO1864-1867) homologous to the *ectABCD *gene cluster required for ectoine biosynthesis in other bacteria was identified in the *S. coelicolor *genome sequence [33]. GO terms for ectoine biosynthesis and production were significantly over-represented in the lists of genes induced following exposure to each of the three antibiotics and the *ectABCD *homologues were up-regulated at all three time points after their addition (see Additional files 1,2 and 3, Additional file 14 Figure 1g). Genes required for gas vesicle biosynthesis were similarly induced, but only those from one of the two *gvp *gene loci, *gvp1 *(Table 1, Additional files 1, 2 and 3, Additional file 14 Figure 1h). A strong induction of *gvp1 *gene expression has also been reported following exposure to high concentrations of salt, and it has been proposed that gas vesicles play a role in the response to hyperosmotic stress [34].

In MIC tests, supplementation of the agar media with 1 mM ectoine provided no significant protection against the effects of either bacitracin or vancomycin (data not shown).

### Changes in expression of genes with functions related to production and maintenance of the cell wall show antibiotic-specificity

#### i) Genes required for cell wall peptidoglycan biosynthesis

**Figure 3:**
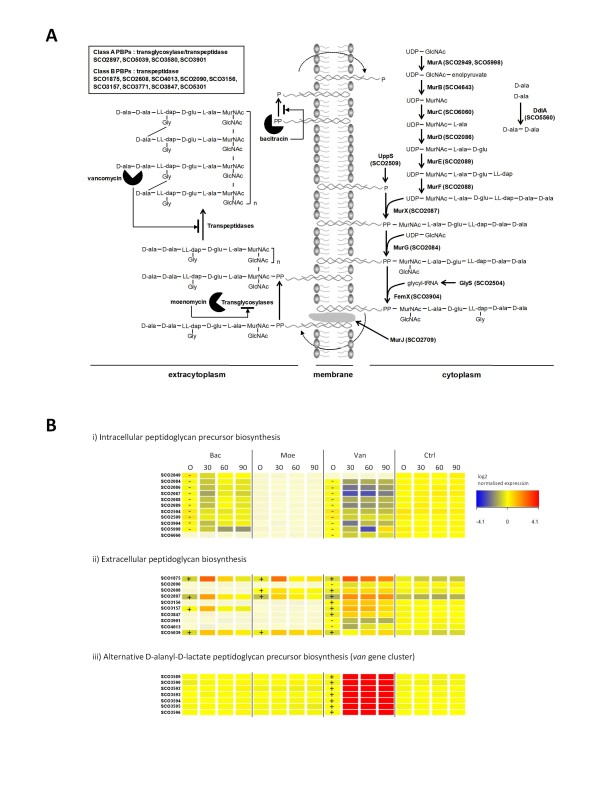
**Bacterial cell wall peptidoglycan biosynthesis and the effects of bacitracin (Bac), moenomycin (Moe) and vancomycin (Van)**. (A) The intracellular and extracytoplasmic steps of peptidoglycan biosynthesis in *S. coelicolor *illustrating the different reactions inhibited by each antibiotic. (B) Heatmap summarising the significant changes in expression of genes involved in bacterial cell wall peptidoglycan biosynthesis following treatment with Bac, Moe or Van. Genes are grouped according to their involvement in the cytoplasmic (i) or extracytoplasmic (ii) stages of biosynthesis illustrated in Figure 3A, or known involvement in the synthesis of alternate cell wall precursors (the *van *gene cluster, Hong et al. [43]) (iii). Genes significantly up- or down-regulated relative to the control (Ctrl) are indicated by a + or -, respectively, and the magnitude of the change is colour coded according to the legend shown. Genes not significantly differently expressed by one of the antibiotics relative to the control are represented by faded boxes.

Bacterial cell wall peptidoglycan biosynthesis can be separated into reactions occurring intracellularly to generate the lipid II precursor subunits, and those taking place extracellularly to join the subunits together into a three dimensional structure [35-42] (Figure 3A). Transcription of many of the genes for the cytoplasmic steps required for precursor biosynthesis, particularly the *mur *operon (SCO2083-2089) and the *femX *gene (SCO3904), were significantly repressed following treatment with either bacitracin or vancomycin, but were unchanged following moenomycin addition (Table 2, Figure 3B). In contrast, genes encoding penicillin-binding proteins (PBPs) required for the extracellular reactions in peptidoglycan production were over-represented in the genes induced by all three antibiotics (Table 1, Figure 3B). The secreted PBPs SCO1875, SCO2897 and SCO5039 were significantly induced following treatment with each compound, while SCO2608 was induced by vancomycin and moenomycin but not by bacitracin. In MIC tests, deletion of SCO1875 markedly increased susceptibility to both bacitracin and vancomycin (Table 3) indicating that this PBP is essential for optimum resistance to these compounds, and suggesting that up-regulation of its transcription is an important response to the antibiotic treatments. Deletion of SCO2897 also increased susceptibility to both antibiotics (lowered the MIC), while mutation in SCO2608 and SCO5039 increased sensitivity to vancomycin only (Table 3). Vancomycin treatment alone down-regulated expression of SCO2090, SCO3901 and SCO4013, and was the only antibiotic to up-regulate transcription of SCO3156 and SCO3847. Consistent with this, deletion of SCO3847 slightly lowered the MIC for vancomycin but showed no effect with bacitracin (Table 3). The PBP encoded by SCO3157 was up-regulated by both vancomycin and bacitracin but not by moenomycin.

The previously characterised *van *gene cluster responsible for remodelling peptidoglcan precursor biosynthesis to produce subunits terminating in D-alanyl-D-lactate (rather than D-alanyl-D-alanine) was, as expected, induced strongly only by vancomycin, and not by either of the other two antibiotics [43,44](Figure 3B). *vanH*, encoding D-lactate dehydrogenase, exhibited the largest change in expression observed for any gene: a 300-fold increase 90 min after vancomycin addition.

#### ii) Genes encoding cell wall remodelling enzymes and sortase substrates

In addition to peptidoglycan biosynthesis, hydrolysis of the cell wall is an important process for growth allowing remodelling of cell shape to enable expansion and differentiation. Haiser et al. [45] identified 57 putative cell wall hydrolase genes in the *S. coelicolor *genome in an *in silico *search, and confirmed that four could efficiently degrade *S. coelicolor *cell walls using biochemical approaches. In this study, genes encoding cell wall hydrolases were significantly over-represented in the lists of genes up-regulated in response to bacitracin or moenomycin, but not vancomycin (see Table 1). In total, expression of 23 of the 57 genes were significantly affected following treatment with one or more of the cell wall antibiotics, only a minority (four) of these however responded in a similar way to all three compounds (Additional file 14 Figure 1e). Seven genes were up-regulated only in response to moenomycin, and four of these were actually repressed following treatment with at least one of the other two antibiotics. In total 13 of the 23 genes were down-regulated in response to either bacitracin or vancomycin, while moenomycin treatment did not negatively affect transcription of any. Interestingly, of the seven members of the resuscitation-promoting factor (Rpf) subgroup of hydrolases, five were significantly induced in response to moenomycin. Only *rpfA *(SCO3097) was induced by all three antibiotics, with expression of the SCO3098 homologue induced by bacitracin and moenomycin, but significantly repressed following vancomycin treatment (Additional file 14 Figure 1e). *rpfA *and SCO3098 encode proteins with putative LysM peptidoglycan-binding domains, and RpfA has been shown to hydrolyse *S. coelicolor *peptidoglycan [45]. Of the three other *rpf *homologues induced by meonomycin, SCO5029 was repressed following bacitracin and vancomycin treatment, while SCO0974 expression was down-regulated by bacitracin. Transcription of SCO3150 was not significantly affected by either vancomycin or bacitracin. The Nlp60-family cell wall hydrolase encoded by SCO4108 was induced in response to both bacitracin and moenomycin, but repressed by vancomycin. Vancomycin and bacitracin strongly induced both SCO5487 (amidase family) and SCO5660 (carboxypeptidase family), while moenomycin up-regulated only the former of these two genes.

Septum synthesis and localisation is a specialised aspect of cell wall biosynthesis important for cell division and sporulation in *S. coelicolor*. SsgA-like proteins are intimately involved in developmental cell division in streptomycetes, and although their precise function has yet to be fully characterised they are believed to control the fate of peptidoglycan during sporulation [46,47]. SsgA-like proteins were significantly over-represented in the vancomycin- and bacitracin-induced gene lists (see Table 1), and *ssgR *(SCO3925), the regulator activating transcription of *ssgA *(SCO3926), was induced 2-to 6-fold at all times following treatment with vancomycin (Additional file 14 Figure 1o). Expression of *ssgD *(SCO6722) was significantly up-regulated by all three antibiotics, while *ssgE *(SCO3158) was induced by vancomycin and moenomycin only.

Sortase enzymes are transpeptidases that covalently link proteins to peptidoglycan in many Gram-positive bacteria [48]. Substrate proteins are recognisable from the presence of amino acid sequence motifs, most notably by a conserved C-terminal LP(A)XTG sequence. Genes encoding 17 substrate proteins were identified in *S. coelicolor *[49], and presumably play a role in cell wall structure and function. For example, three are known to encode chaplin proteins that assemble as a hydrophobic coat during aerial mycelium formation when cultured on solid agar media [50]. Genes encoding sortase substrates were significantly over-represented in the lists of genes repressed by each of the drugs (see Table 2), with six genes significantly altered in expression following treatment with one or more of the antibiotics (Additional file 14 Figure 1n). Four (SCO2492, SCO2682, SCO3176 and SCO5650) were down-regulated in comparison to the control experiment by all three compounds, while SCO2270 was repressed only by bacitracin and vancomycin. SCO1023, encoding a protein of unknown function, was induced in response to all three antibiotics. As expected, expression of the chaplin genes was not significantly altered since cells were grown in submerged liquid culture where aerial mycelium formation does not take place.

### The biosynthesis of antibiotics is induced in response to cell wall specific drugs

**Figure 4:**
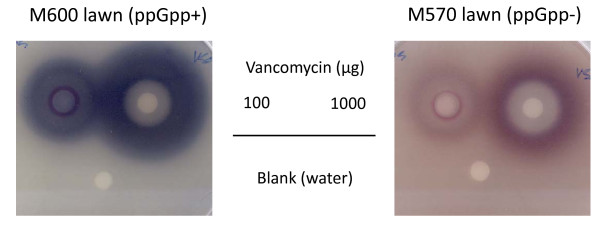
**Exposure to vancomycin induces biosynthesis of the pigmented antibiotics Act and Red in *S. coelicolor***. This response is markedly reduced in strain M570 that is defective in production of the stringent factor ppGpp. Spore lawns of the indicated strains were spread on SMMS agar plates [81], and paper discs impregnated with vancomycin (or water) were applied to the surface. Cultures were incubated for 3 days at 30°C.

In the gene ontology analysis (Additional files 5, 6,7, 8, 9, 10 and 11), the GO term for acyl carrier activity was prominent in the lists from the term-for-term and MGSA analysis of genes induced by vancomycin or bacitracin in the 90 min sample, with as many as 8 genes associated with this term present in the lists of up-regulated genes. These correspond to four genes each from the biosynthetic clusters responsible for producing the antibiotics CDA and undecylprodiginine (Red), and indicate that activation of antibiotic production is part of the cellular response to exposure to both vancomycin and bacitracin. Indeed, 39 of the 47 CDA cluster probe sets (corresponding to 32 of the 40 genes) were up-regulated after addition of vancomycin, along with all 23 Red cluster probe sets (22 of the 22 genes; Table 1, Additional files 14 Figures 1d and 1i). The corresponding figures for bacitracin treatment were 35 and 9 probe sets, respectively, while moenomycin induced 11 probe sets from the CDA cluster. Activation of CDA synthesis is therefore a common response to treatment with all of the antibiotics. The expression of the CDA and Red biosynthesis genes, and that of the only other antibiotic biosynthetic gene cluster identified in the genome, the actinorhodin (Act) cluster, is ultimately dependent on activation by regulatory proteins encoded by each cluster [51-53]. The changes in expression of these activator genes brought about by treatment with the cell wall antibiotics is summarised in Table 4, and indicates that vancomycin and bacitracin in particular had a strong inducing effect on transcription of all four activators. Although an increase in expression of the actinorhodin biosynthesis genes was not observed in the 90 min in liquid culture following addition of any of the antibiotics, vancomycin treatment induced production of the blue pigmented antibiotic in a paper disc assay using growth on a solid agar medium (Figure 4). This response was markedly reduced, but not abolished, in an M570 mutant defective in ppGpp synthesis, indicating that the signalling molecule is not essential for this response to vancomycin.

**Table 4:** Expression of the genes encoding the transcriptional activators for switching on production of the CDA, Red and Act biosynthetic clusters is induced following exposure to vancomycin, bacitracin and moenomycin.

Antibiotic cluster	Activator gene vancomycin	Fold-change increase in expression in the 90 min sample^1^		
		
		Vancomycin	Bacitracin	Moenomycin
CDA	*cdaR *(SCO3217)	5.4	2.4	1.5
Act	*actII*-ORF4 (SCO5085)	2.8	1.6	1.5
Red	*redD *(SCO5877)	11.4	4.4	2.1
Red	*redZ *(SCO5881)	5.7	3.6	NA

^1^NA = the gene was not present in the list of significantly differently expressed genes in Additional files 1, 2 and 3.

### Sigma factor gene expression is a major response to exposure to cell wall antibiotics

*S. coelicolor *RNA polymerase can interact with any one of 64 different sigma factors, each specifying recognition of a different cognate consensus promoter sequence [54]. Changes in the relative abundance of sigma factors within a cell can therefore significantly influence the pattern of gene transcription. Sigma factor genes were significantly over-represented in the lists of genes induced by each antibiotic (Table 1) indicating that alteration in sigma factor gene expression is an important response to treatment with antibiotics targeting cell wall biosynthesis. This is also reinforced by the prominence of genes known to be regulated by σ^E^, σ^B ^and σ^R ^in the differentially expressed gene lists (see Tables 1 and 2), and by the observed increase in susceptibility of σ^E ^and σ^B ^mutant strains towards bacitracin and vancomycin (see Table 3). Eight sigma factor genes, including σ^E ^(SCO3356) and σ^B ^(SCO0600), were up-regulated following treatment with all three compounds. Of these, the genes encoding the putative extra-cytoplasmic function (ECF) sigma factors encoded by SCO4005, SCO4908 and SCO5147 were particularly strongly induced (5- to 7-fold by vancomycin; Additional file 14 Figure 1l). A mutant strain in which SCO4005 was disrupted by insertion of a transposon showed increased susceptibility to bacitracin, but was not significantly altered in its resistance to vancomycin (Table 3). However, a similar mutant in which SCO5147 had been disrupted showed no increase in susceptibility to either compound and was slightly more resistant to vancomycin than the parental strain (Table 3). Transcription of the alternative sigma factor σ^hrdD ^(SCO3202) known to be dependent on σ^E ^[20] was also up-regulated by all three antibiotics, while the expression of σ^bldN ^(SCO3323), a sigma factor important for *S. coelicolor *differentiation [55], was repressed by all three. Vancomycin alone repressed transcription of four other sigma factor genes (SCO3068, SCO3626, SCO5216 (σ^R^), and SCO5243); two of these, including σ^R^, were up-regulated after treatment with at least one of the other two antibiotics. Consistent with this, genes from the σ^R^-regulon were significantly over-represented in both the vancomycin-repressed and moenomycin-induced gene lists (see Tables 1 and 2).

### The zinc-responsive *zur *regulon is induced exclusively by vancomycin

Zinc plays an indispensable role in cellular biochemistry as a catalytic or structural cofactor for a wide variety of metalloproteins. It can however be toxic if accumulated to excess, and its intracellular concentration is controlled within safe limits by homeostatic regulatory mechanisms. In bacteria this is largely achieved by tight control of import and export mechanisms via the zinc-responsive regulatory protein Zur [56,57], and the Zur regulon in *S. coelicolor *has recently been characterised [58-60]. Interestingly in this work the majority of the genes in the regulon, including the *znuABC *operon (SCO2505-2507) encoding the high-affinity Zn^2+ ^import system, were transiently up-regulated following addition of vancomycin but not by either of the other two antibiotics (Table 1, Additional file 14 Figure 1p). Expression of *znuA *(SCO2505) was induced 12-fold 30 min after vancomycin addition, decreasing to about 2-fold up-regulated by 90 min (see Additional file 3).

### Genes required for branched chain amino acid and biotin biosynthesis are induced by moenomycin

GO terms associated with amino acid biosynthesis were over-represented in the genes induced in response to moenomycin treatment (see Additional files 5, 6, 7, 8, 9 and 10). Comparison of the significantly differently expressed genes with the groups of functionally related genes in Tables 1 and 2 supported this observation, indicating that moenomycin induced genes associated with branched chain amino acid (leucine, isoleucine and valine), lysine and methionine biosynthesis. Indeed, the *ilvD *(SCO3345), *leuB *(SCO5522) and *leuC *(SCO5553) genes for the biosynthesis of the branched chain amino acids leucine, valine and isoleucine were up to 7-fold up-regulated by moenomycin treatment (Additional file 14 Figure 1c). In addition, genes associated with the transport of amino acids were over-represented in both the moenomycin up-regulated genes, and those down-regulated by the antibiotic (Tables 1 and 2). Interestingly, the down-regulated genes included four from the putative branched-chain amino acid import operon SCO2008-2012 (Additional file 14 Figure 1a).

GO analysis of the 252 genes specifically up-regulated by moenomycin in Figure 1B indicated a significant over-representation not only of amino acid biosynthesis genes, but also of biotin biosynthesis. The *bioB-D *operon (SCO1244-1246) was approximately 2-fold up-regulated in the 90 min moenomycin-treated sample. Biotin is a cofactor implicated in catabolism of fatty acids and branched chain amino acids, and up-regulation of its production may indicate an increased use of branched chain amino acids as an energy source in the moenomycin treated cells. Indeed, expression of *accD1 *(SCO2776) encoding the biotin-dependent acetyl-CoA carboxylase enzyme, a key enzyme in branched chain amino acid degradation, was 5-fold up-regulated 90 min after moenomycin addition (see Additional file 3).

### Genes encoding two-component signal transduction systems are specifically induced by bacitracin

GO analysis of the 39 genes whose transcription was induced only in response to bacitracin (see Figure 1B) indicated genes associated with two-component system signal transduction as the most over-represented process (see Additional file 11), with ten of the genes encoding response regulator or sensor kinase proteins. These included two clusters of three genes encoding a response regulator-kinase-kinase (SCO4596-4598), and a regulator-regulator-kinase (SCO3638, SCO3640 and SCO3641). This implies that they represent multi-component signal transduction systems that are important for coordinating a response to bacitracin (but not moenomycin or vancomycin). Other two-component system genes uniquely up-regulated in response to bacitracin were the SCO5683-5684 regulator-kinase pair, and the kinases encoded by SCO2359 and SCO7649. Mutant strains deleted for SCO3638-3641, SCO4596-4598 or SCO5683-5684 did not however show any significant change in their resistance to bacitracin (data not shown).

### Identification of novel ABC transport systems important for bacitracin resistance

**Figure 5:**
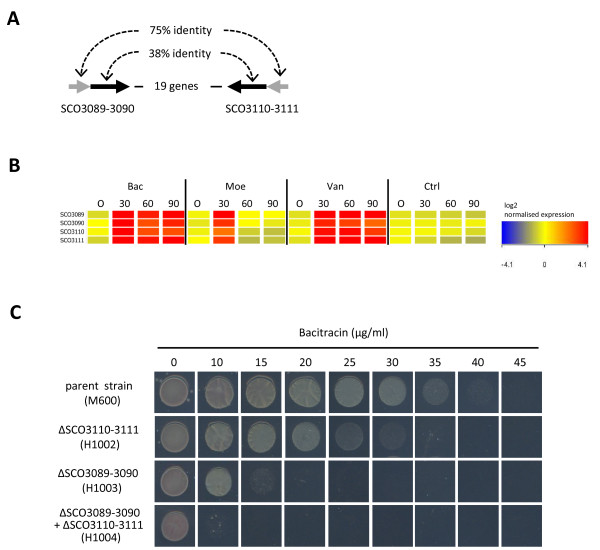
**Identification of novel ABC transport systems important for bacitracin resistance**. A) Homologous pairs of genes encoding putative ABC transport integral membrane (black arrow) and ATP-binding (grey arrow) proteins located close to each other on the chromosome. B) Transcription of both transport systems is strongly up-regulated following treatment with bacitracin (Bac), moenomycin A (Moe) and vancomycin (Van). Heatmap summarising the significant changes in expression of the genes relative to the control (Ctrl). The magnitude of the change in expression is colour coded according to the legend shown. C) Deletion of the transport systems individually or together increases susceptibility to bacitracin.

SCO3089-3090 and SCO3010-3011 encode putative ABC transport proteins that show a high degree of homology: SCO3089 is 75% identical to SCO3111 at the amino acid level, while SCO3090 shows 38% identity over its full-length to SCO3110 (Figure 5A). Transcription of all four genes was strikingly up-regulated 30-fold or higher in response to each of the three compounds (Figure 5B, Additional files 1, 2 and 3), suggesting an important role for these transporters in the response to the antibiotic treatments. To test this further, mutant strains carrying deletions in one or both of the transport systems were constructed and analysed for their susceptibility to vancomycin and bacitracin (Table 3 and Figure 5C). While the single and double mutant strains showed at most only a marginal increase in susceptibility to vancomycin, the double mutant strain H1004 exhibited markedly increased susceptibility to bacitracin, reducing the MIC to below 10 μg/ml compared to 40-45 μg/ml for the parental strain. Both single mutant strains also showed an increased susceptibility to bacitracin, but MIC values (15-20 μg/ml for H1003 (ΔSCO3089-3090) and 30-35 μg/ml for H1002 (ΔSCO3110-3111)) were not as low as for the double mutant.

## Discussion

### Stress response genes contribute towards antibiotic resistance in *S. coelicolor*

Vancomycin, bacitracin and moenomycin target distinctly different stages of cell wall biosynthesis (see Figure 3A), yet exposure of *S. coelicolor *to each antibiotic resulted in changes in expression of many of the same genes (see Figure 1). Previous studies on the role of the ECF (extracytoplasmic function) sigma factor σ^E ^in maintaining cell wall homeostasis in *S. coelicolor *identified a key role in responding to antibiotics that damage the cell wall [19]. This study reveals that the transcriptional response to vancomycin, moenomycin and bacitracin extends far beyond the involvement of σ^E^, incorporating elements of the heat shock response, the stringent response, and the response to osmotic and oxidative stress. Whether this reflects different, condition-specific regulatory cascades influencing the same genes, or triggering of the same regulatory cascade by different stimuli is unclear. The failure of vancomycin to increase synthesis of the stringent factor ppGpp while modulating expression of many stringently controlled genes suggests the former, but simultaneous induction of transcription of σ^B ^and the σ^B ^regulon, and of σ^E ^and the σ^E^-dependent gene SCO3202, by all three antibiotics would be indicative of the latter. A combination of the two appears likely. These stress responses were not necessary for antibiotic resistance, but were required for maximal resistance. A σ^B^-deletion mutant strain and a mutant defective in ppGpp synthesis both exhibited increased susceptibility towards bacitracin and vancomycin. *S. coelicolor *carries an inducible system that specifically confers high-level (MIC ~80 μg/ml) resistance to vancomycin, and functions by reprogramming cell wall precursor biosynthesis [43,44]. Although the *van *genes involved are among the most highly induced in response to vancomycin treatment at all time points tested (see Figure 3B, Additional file 1), it is unlikely that cells become instantly fully resistant to the effects of the antibiotic and expression of the stress response genes may facilitate survival during this transition period. A transient slowing in growth was observed following treatment with both vancomycin and bacitracin, consistent with the existence of such a "stress" phase (data not shown).

*S. coelicolor *is susceptible to bacitracin (MIC 40-45 μg/ml) but highly resistant to moenomycin (MIC >300 μg/ml). All of the mutants tested were also resistant to 300 μg/ml moenomycin. It is perhaps therefore not surprising that moenomycin repressed the transcription of many fewer genes than either bacitracin or vancomycin (see Figure 1A). Interestingly however, moenomycin activated the transcription of a comparable number of genes to both vancomycin and bacitracin, including genes from the heat shock regulon, the stringent response, and the osmotic and oxidative stress regulons. Treatment with 10 μg/ml moenomycin, although only a fraction of the MIC, clearly induced a physiological response, perhaps reflecting induction of mechanisms for cell wall homeostasis well below inhibitory concentrations of the antibiotic or even a signalling role for the compound (see below).

### Identification of new genes with roles in antibiotic resistance

Genes whose transcription was activated in response to two or more of the compounds may be components of a general response to treatment with antibiotics inhibiting cell wall biosynthesis. Inactivation of two putative ABC transporters, SCO3089-3090 and SCO3110-3111, both markedly up-regulated after treatment with all three antibiotics, significantly increased susceptibility to bacitracin. The integral membrane proteins encoded by these two systems are similar to lysophospholipase L1 and transport system components associated with lipoprotein release (http://streptomyces.org.uk), possibly suggesting a role in remodelling the cell envelope. Interestingly, orthologous and syntenous systems also occur in the genomes of *Streptomyces clavuligerus*, *Streptomyces avermitilis *and *Streptomyces griseus*, suggesting conserved evolution and a common biological function. The ECF sigma factor encoded by SCO4005 also contributes to bacitracin resistance; identifying the SCO4005 regulon could provide new insights into how bacterial cells cope with the antibiotic.

Of the other genes inactivated and analysed for their effect on antibiotic resistance, only disruption of the PBP encoded by SCO1875 and of the putative lysyl-tRNA synthetase encoded by SCO3397 markedly increased susceptibility to both bacitracin and vancomycin. Transcription of both SCO1875 and SCO3397 is markedly up-regulated following treatment with each of the antibiotics and is therefore an important part of the antibiotic resistance response. Navratna et al. [61] reported that deletion of the gene encoding PBP4 in *Staphylococcus aureus *increased susceptibility to vancomycin and was associated with a reduction in cross-linked muropeptide. PBP5 in *Enterococcus faecalis *is required for resistance to cephalosporins [62]. None of these three PBPs are essential for growth in their respective organisms, yet modulation of their activities appears to be an important component of the response to compounds that inhibit peptidoglycan biosynthesis. The response to vancomycin treatment included the up-regulation of transcription of eight genes encoding putative PBPs, including two only induced by this antibiotic. These observations, together with three PBPs genes uniquely repressed following vancomycin addition are discussed later. Of the 28 genes predicted to encode tRNA synthetases in the *S. coelicolor *genome, only SCO3397 was up-regulated following drug treatment, the majority (21) typically being down-regulated. In addition to their central role in protein biosynthesis, aminoacyl-tRNAs are involved in porphyrin biosynthesis, N-terminal protein modification, peptidoglycan biosynthesis and aminoacyl-phosphotidylglycerol biosynthesis [63]. SCO3397 is required for maximal resistance to bacitracin and vancomycin in *S. coelicolor*, and homologous MprF proteins in *Staphylococcus aureus *and *Listeria monocytogenes *have also been implicated in resistance to antimicrobial peptides [64,65]. Although it is not known whether SCO3397 possesses phospholipid lysylination activity similar to MprF, this protein class may represent a useful target for counteracting resistance to antibiotics active against the cell envelope.

### Genes responding uniquely to individual drug treatments offer new insights into activity and resistance

While there were many similarities in the changes in gene expression following treatment with vancomycin, moenomycin or bacitracin, genes responding uniquely to only one of the antibiotics were also identified (see Figure 1B). Given the different activities of the three compounds, these results potentially provide new insights into the mode of action of, and mechanisms of resistance towards, the individual antibiotics.

#### i) Vancomycin

In total, transcription of 235 genes was uniquely activated by vancomycin, while 454 were uniquely repressed (see Figure 1B). As expected, the genes uniquely up-regulated included the set of seven *van *genes previously characterised as encoding an inducible resistance system specific to this drug [43,44]. Surprisingly, they also included 19/22 genes from the Zur regulon required for zinc homeostasis [58-60] that were transiently but strongly up-regulated in the 30 min sample. The implication is that vancomycin addition caused a decrease in intracellular zinc availability that was subsequently corrected via the Zur-dependent zinc starvation response. How vancomycin causes zinc limitation is not known, but could result from changes in the cell envelope that favour zinc sequestration or perhaps via chelation of zinc by the antibiotic itself (vancomycin interacts with Cu^2+ ^ions *in vitro *[66,67]). Transcription of five genes encoding PBPs and therefore with putative roles in extracellular peptidoglycan biosynthesis were uniquely significantly affected by vancomycin treatment (SCO3156 and SCO3847 were up-regulated while SCO2090, SCO3901 and SCO4013 were repressed). It is interesting to speculate that at least some of these changes may be associated with the reprogrammed peptidoglycan biosynthesis producing precursors terminating in D-alanyl-D-lactate and perhaps requiring PBPs to match the changes in substrates. SCO5034-5035 encoding a putative ABC transporter was 3.5-fold or more up-regulated at all time points in response to vancomycin, but not moenomycin or bacitracin (see Additional file 14, Figure 1b). The products of these genes are homologous to the transporters encoded by SCO3089-3090 and SCO3110-3111 (about 20% identity between the permease proteins, and 40% between the ATP-binding subunits) that are strongly induced in response to all three antibiotics. Functional redundancy associated with vancomycin-dependent expression may explain why the double mutant strain H1004 (ΔSCO3089-3090 + ΔSCO3110-3111) was sensitive to bacitracin but not to vancomycin. Interestingly, the putative regulatory gene SCO5036 immediately upstream of SCO5034-5035 was also induced by vancomycin, and was >15-fold up-regulated at all times following antibiotic treatment. From their chromosomal arrangement these genes appear to form a single transcription unit directed from a promoter upstream of SCO5036.

#### ii) Bacitracin

Genes exclusively induced by bacitracin are particularly interesting because none correspond to previously known bacitracin resistance genes in other organisms, such as *bacA*, *bcrA*, and *bcrC *[68-72]. This suggests that *S. coelicolor *may possess a novel mechanism for bacitracin resistance. Treatment with bacitracin activated the transcription of many two-component regulatory systems, including several whose strong induction was unique to this antibiotic. In fact, about 25% of the genes uniquely up-regulated in response to bacitracin treatment encoded response regulators or sensor histidine kinases. However, mutant strains carrying deletions in individual sensory systems showed no change in susceptibility to bacitracin, indicating, in contrast to vancomycin, the existence of multiple, functionally redundant sensory systems. The *bcrA *and *bcrC *homologues induced by bacitracin in this work were also similarly up-regulated by at least one of the other two antibiotics, and are therefore likely to be involved in a generalised antibiotic response. Transcription of the *bacA *homologue SCO7047 was not significantly affected by any of the antibiotics.

#### iii) Moenomycin A

Moenomycin A exposure was the only treatment that activated transcription of genes required for amino acid biosynthesis; vancomycin and bacitracin tended to repress their expression. Genes encoding enzymes with roles in branched chain amino acid biosynthesis were particularly strongly induced, while those required for their import were simultaneously repressed. While this may reflect inhibition by moenomycin of the uptake of these amino acids, it could also indicate an increase in catabolism of branched chain amino acids. The latter is supported by a simultaneous and moenomycin-specific up-regulation in the catabolic enzyme *accD1 *and in genes required for the biosynthesis of biotin, an important cofactor for catabolic processes. Branched chain amino acids are important precursors in secondary metabolite biosynthesis, and their availability can affect secondary metabolite production in *S. coelicolor *[73]. Preferential use of these precursors for energy yielding catabolic processes in the moenomycin-treated cells could potentially explain the lack of activation by moenomycin of the secondary metabolic pathways for CDA and Red.

### Induction of antibiotic biosynthesis following antibiotic treatment: chemical warfare in the soil?

*S. coelicolor *M600 produces three antibiotics, actinorhodin (Act), undecylprodiginine (Red) and the calcium-dependent antibiotic CDA. Strikingly, expression of the regulatory genes required for activating transcription of all three biosynthetic gene clusters was up-regulated within 30-60 min of exposure to both vancomycin and bacitracin, and was accompanied by a marked increase in transcription of the Red and CDA biosynthesis genes. Moenomycin also induced the CDA biosynthetic gene cluster. Production of the pigmented antibiotics Act and Red in response to vancomycin treatment was readily detectable in an agar disc assay (see Figure 4). *S. coelicolor *shares its native soil environment with a host of other microbial strains capable of secreting their own antibiotic metabolites into their immediate surroundings, and it appears to have evolved to respond by activating production of its own bioactive metabolites. Whether it does so as a form of retaliatory chemical warfare or as a means of inter-species communication is open to speculation.

### Antibiotics as signalling molecules?

The production of metabolites with antibiotic activity is a widespread trait of soil microorganisms, and must confer some evolutionary advantage. While some of these compounds may indeed be used to inhibit competing species, they may also act as signalling molecules, particularly at sub-inhibitory concentrations [74-77], where they can modulate the transcriptional profiles of other bacteria [78-80]. The significant transcriptional response of *S. coelicolor *to treatment with moenomycin at levels corresponding to <5% of the MIC may represent another such example.

## Conclusions

This work has used the novel approach of identifying common and unique transcriptional changes following the treatment of bacteria with three antibiotics that share the same key cellular target (cell wall biosynthesis) but differ at the molecular level in their mode of action. The characterised responses provide a wealth of information pertinent to generalised and antibiotic-specific cellular processes important for resistance and adaptation to the compounds used. Regulatory networks known to govern responses to environmental and nutritional stresses were shown to be at the heart of the common antibiotic response, and likely help cells survive until any specific resistance mechanisms are fully functional. Attenuation of these networks by mutation increased susceptibility to antibiotic treatment. Genes with putative roles in the extracellular synthesis and remodelling of peptidoglycan exhibited antibiotic-specific transcriptional changes, as did genes encoding two-component sensor systems and sigma factors. Antibiotic susceptibility studies using mutants constructed on the basis of the transcriptome profiling confirmed a role for several genes in antibiotic resistance, validating the usefulness of the approach. This included genes encoding two novel ABC transport systems required for bacitracin resistance, and a penicillin-binding protein and lysyl-tRNA synthetase that contribute to both bacitracin and vancomycin resistance. These represent new leads for further improving our understanding of how bacteria resist antimicrobial treatments at the molecular level, knowledge which may well be vital in enabling the future development of more effective antibiotics.

## Materials and methods

### Bacterial strains and culture conditions

*S. coelicolor *M600, a prototrophic plasmid-free derivative of *S. coelicolor *A3(2), was used for the microarray study. Spores of *S. coelicolor *M600 were germinated and grown to mid-log phase (OD600 nm approx. 0.5) in NMMP as described previously [19,43,44]. Biological triplicate cultures were treated at this point by adding a sub-lethal concentration (10 μg/ml) of vancomycin, bacitracin or moenomycin A and samples taken 0, 30, 60, 90 minutes after addition of the antibiotic. A negative control which received no antibiotic treatment was also performed. All of the antibiotics used for this study except moenomycin A were purchased from Sigma-Aldrich. Purified moenomycin A was a kind gift from Professor Peter Walzel in the University of Leipzig. All strains used are listed in Table 3.

Paper disc assays were used to study the induction of pigmented antibiotics by vancomycin. Spore lawns of M600 and M570 were spread on SMMS agar plates [81], and paper discs impregnated with vancomycin (or blank) were immediately applied to the surface. Cultures were incubated for 3 days at 30°C.

### RNA isolation and DNA microarray analysis

RNA was isolated from liquid cultures according to Hesketh et al. (2007) [21]. Purified total RNA (10 μg) was processed into labelled and fragmented cDNA for hybridisation to *Streptomyces *diS_div712a Affymetrix GeneChip arrays as previously described [21]. Following scanning of the arrays, the data quality was verified using a variety of tools including the 'affyPLM', 'affy' and 'simpleaffy' packages for the statistical computing environment R [82], quality control methods available within GeneSpring 11 (Agilent), and data from report files generated in the Affymetrix Genechip Operating Software. One replicate from the bacitracin experiment failed quality control and was excluded from further analysis, leaving duplicate data for this antibiotic treatment. All microarray data are available from ArrayExpress (http://www.ebi.ac.uk/arrayexpress/) under accession number E-MEXP-3032.

To identify differentially expressed genes, array data were first imported into GeneSpring 11 with normalisation using the Robust Multichip Average (RMA) algorithm of Irizarry et al. [83]. The data were then filtered to remove genes deemed to be expressed at a level below reliable quantification by determining those with a raw signal value below a defined background cut-off value of 100 in all samples. The results were further filtered to remove genes deemed to be unresponsive to the conditions under test in the experiment by identifying those with normalised expression values between 0.667-1.5 (1.5-fold change limit) in all conditions. The filtered data (2789 genes) were then subjected to two-way ANOVA to identify genes significantly altered under the experimental conditions, contrasting the expression values in each antibiotic-treated sample with the corresponding time point from the untreated control. This was performed using the parametric test option with a false discovery rate of P < 0.01. P-values were corrected using the Benjamini and Hochberg false discovery rate multiple testing correction procedure, and were computed asymptotically. Details of the statistical calculations used in the software can be accessed through the manufacturer's manual.

Gene ontology (GO) analysis was performed on lists of differentially expressed genes to identify over-represented biological functions or processes using the *S. coelicolor *GOA annotation from EMBL-EBI (ftp://ftp.ebi.ac.uk/pub/databases/GO/goa/proteomes/84.S_coelicolor.goa) as listed on 17/12/2009. GO analyses were realised using the Ontologizer tool (http://compbio.charite.de/index.php/ontologizer2.html), performing term-for-term hypergeometric testing with the Benjamini and Hochberg false discovery rate correction, or a Bayesian modelling approach using the model-based gene set analysis (MGSA) option [84]. Statistical comparison of lists of differentially expressed genes to in-house curated lists of functionally related genes was performed using the 'find similar entity lists' tool of Genespring 11. The results (summarised in Tables 1 and 2) determined whether the number of genes from a particular functional class were over-represented in the data, and therefore biologically meaningful, or could have occurred by chance. Absence of a functional class in Tables 1 and 2 therefore does not mean that all genes in that class are absent in the data, but rather that the number of genes present could have arisen simply by chance at the 5% probability level used. Quality threshold (QT) clustering was performed in Genespring 7.3.

### Construction of mutant strains for determining their antibiotic susceptibility

Targeted gene deletions were constructed using the PCR-directed mutagenesis procedure of Gust et al. [85] or by using transposon mutated cosmids from the library of Bishop et al. [86]. Briefly, for PCR-directed replacement of genes with an antibiotic resistance cassette (e.g. *aac(3)IV *for apramycin resistance, *hyg *for hygromycin resistance), cosmid DNA in *E. coli *was targeted with a disruption cassette created by PCR using gene-specific primers and pIJ773 (for *aac(3)IV*) or pIJ10007 (for *hyg*) as template. Candidate antibiotic resistant clones were verified by PCR and restriction digestion, and conjugated into *S. coelicolor *M600. Double crossover integrants were selected as apramycin/hygromycin resistant, kanamycin sensitive clones, and verified by PCR. For transposon mutants, mutated cosmids from the library were conjugated into *S. coelicolor *M600 and double crossover integrants were selected as apramycin resistant, kanamycin sensitive clones.

### Assay for determining the minimum inhibitory concentration (MIC) of the antibiotics

Minimum inhibitory concentration (MIC) values were evaluated using a method similar to that of Andrews (2001) [87]. McFarland turbidity standards of 0 (1.0 ml distilled water), 0.5 (5.0 μl 1.175% BaCl_2_, 995 μl 1% H_2_SO_4_), 1 (10.0 μl 1.175% BaCl_2_, 990 μl 1% H_2_SO_4_), and 2 (20 μl 1.175% BaCl_2_, 980 μl 1% H_2_SO_4_) were prepared. *S. coelicolor *spores from 20% glycerol stocks were added to 1.0 ml distilled water until the turbidity of the spore suspension was between McFarland standards 1 and 2. Spore suspensions were stored at 4°C and used within 1 week, during which time they showed no loss of viability. 5.0 μl aliquots of the spore suspensions were spotted onto a series of MMCGT agar plates [19] containing the antibiotic under test at a range of concentrations (vancomycin 30-150 μg/ml in 10 μg/ml increments; bacitraicin 10-45 μg/ml in 5 μg/ml increments; moenomycin 0-300 μg/ml in 20 and 50 μg/ml increments). The spots were allowed to dry, and the plates were incubated at 30°C. MIC values were determined by visual inspection of growth over the range of antibiotic concentrations after 48 hrs incubation.

### Analysis of intracellular nucleotide pools

Quantification of intracellular nucleotides was performed by HPLC as described in Hesketh et al. [21]. Duplicate cultures of *S. coelicolor *grown to mid-exponential phase (OD600 nm approx. 0.5) and treated with 10 μg/ml vancomycin were extracted and analysed at 0, 15 30 and 60 min following addition of the antibiotic.

## Abbreviations used

Act: actinorhodin; ANOVA: analysis of variance; CDA: calcium-dependent antibiotic; ECF: extra-cytoplasmic function; GO: gene ontology; MGSA: model-based gene set analysis; MIC: minimum inhibitory concentration; MMCGT: minimal medium supplemented with casamino acids, glucose, tiger milk; MRSA: methicillin resistant *Staphylococcus aureus*; PBP: penecillin-binding protein; Red: undecylprodiginine

## Competing interests

The authors declare that they have no competing interests.

## Authors' contributions

HJH conceived and designed the study, performed the microarray experiments, assisted with data analysis and interpretation, and lead the writing of the manuscript. AH assisted with the microarray experiments, performed the microarray data analysis and interpretation, drafted the manuscript, and contributed to the construction and phenotypic characterisation of mutant strains. MB participated in the experimental design, and contributed to the writing of the manuscript. CH, JM, GN and NT all contributed to the construction and phenotypic characterisation of mutant strains. All authors read and approved the final manuscript.

## Supplementary Material

Additional file 1 **Summary of transcriptome analysis of genes significantly differently expressed in response to vancomycin treatment**. Click here for file

Additional file 2 **Summary of transcriptome analysis of genes significantly differently expressed in response to bacitracin treatment**. Click here for file

Additional file 3 **Summary of transcriptome analysis of genes significantly differently expressed in response to moenomycin treatment**. Click here for file

Additional file 4 **Normalised expression data for the differentially expressed gene groups illustrated in the venn diagram of Figure **1B. Click here for file

Additional file 5 **Term for term GO analysis of the genes significantly differently expressed in response to vancomycin**. Click here for file

Additional file 6 **Term for term GO analysis of the genes significantly differently expressed in response to bacitracin**. Click here for file

Additional file 7 **Term for term GO analysis of the genes significantly differently expressed in response to moenomycin**. Click here for file

Additional file 8 **MGSA GO analysis of the genes significantly differently expressed in response to vancomycin**. Click here for file

Additional file 9 **MGSA GO analysis of the genes significantly differently expressed in response to bacitracin**. Click here for file

Additional file 10 **MGSA GO analysis of the genes significantly differently expressed in response to moenomycin**. Click here for file

Additional file 11 **Term for term GO analysis of the differentially expressed gene groups illustrated in the venn diagram of Figure **1B. Click here for file

Additional file 12 **Analysis of the differentially expressed gene groups illustrated in the venn diagram of Figure **1B, **looking for significant similarity with in-house curated lists of functionally related genes**. Click here for file

Additional file 13 **Genes whose expression following treatment with all three antibiotics is closely correlated (Pearson correlation > 0. 9) with transcription of the cell wall stress sigma factor σ^E ^(SCO3356)**. Click here for file

Additional file 14 **Heatmaps summarising the expression of genes significantly differently expressed in response to drug treatment, grouped according to related function**. Click here for file
