# Supplementary material for: Genome-wide dynamics of a bacterial response to antibiotics that target the cell envelope
Source: BMC Genomics. 2011 May 11;12:226. doi: 10.1186/1471-2164-12-226 (PMC3123327; doi:10.1186/1471-2164-12-226)

Supplementary Fig. 1

Heatmaps summarising the expression of genes significantly differently expressed in response to drug treatment, grouped according to related function. Up- or down-regulation is indicated by a + or -, respectively,. Genes not differently expressed by one of the drugs relative to the control have been whited out.

a) Amino acid transport

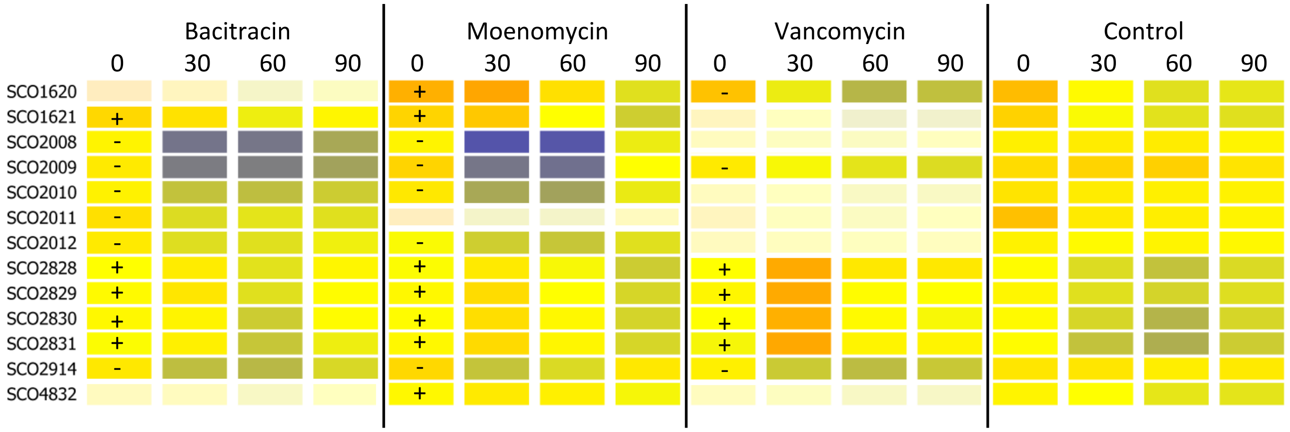

b) ABC transport

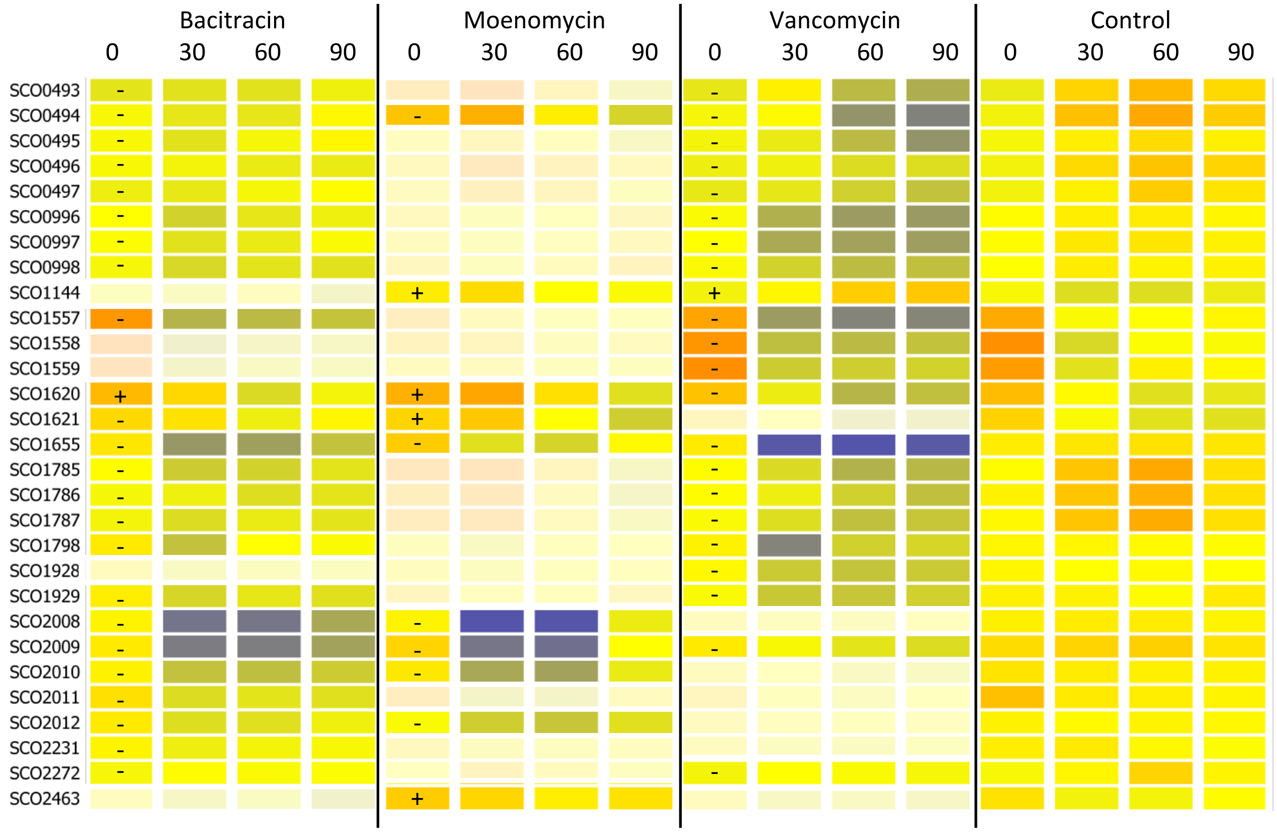

b) ABC transport (continued)

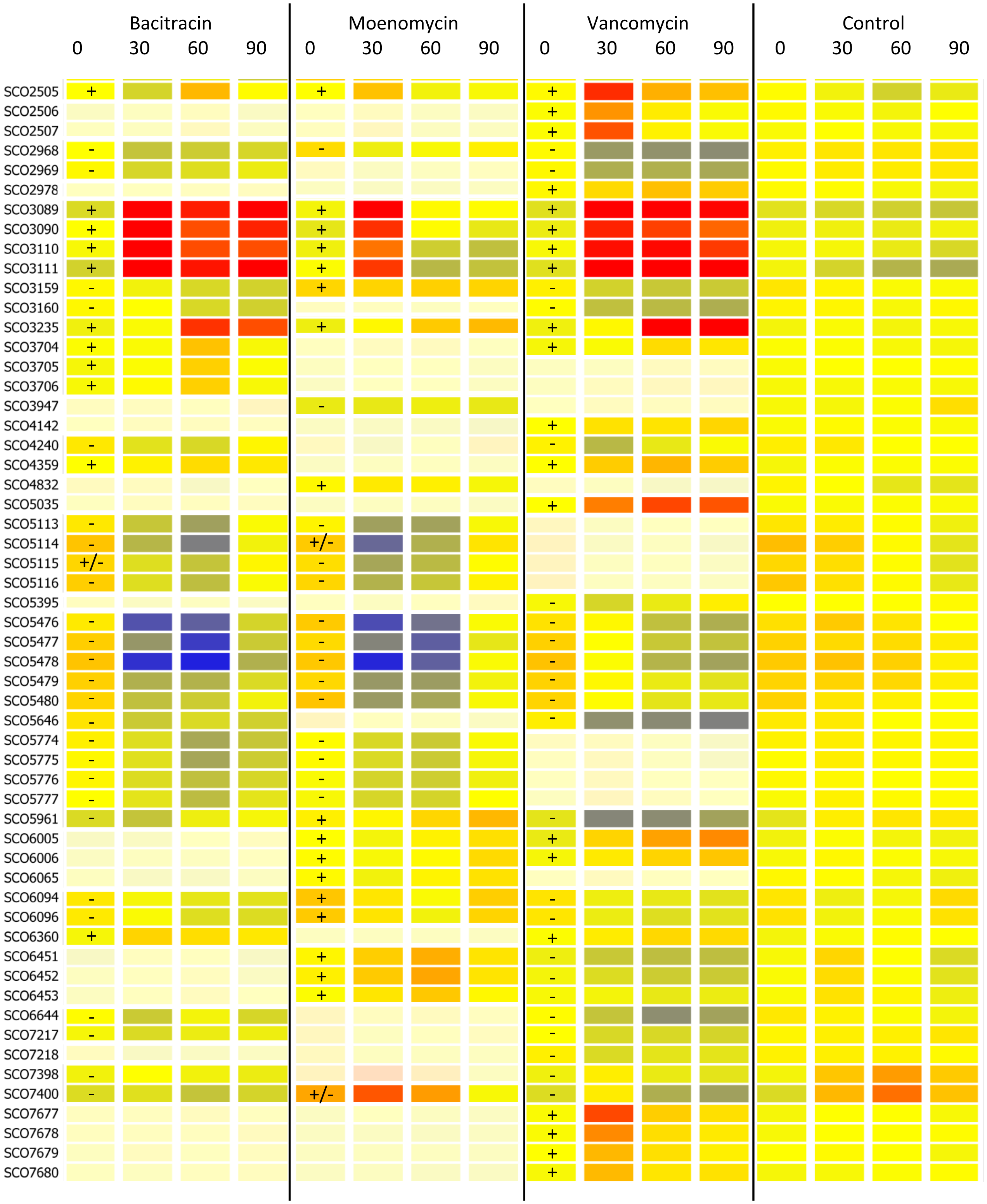

c) Amino acid biosynthesis

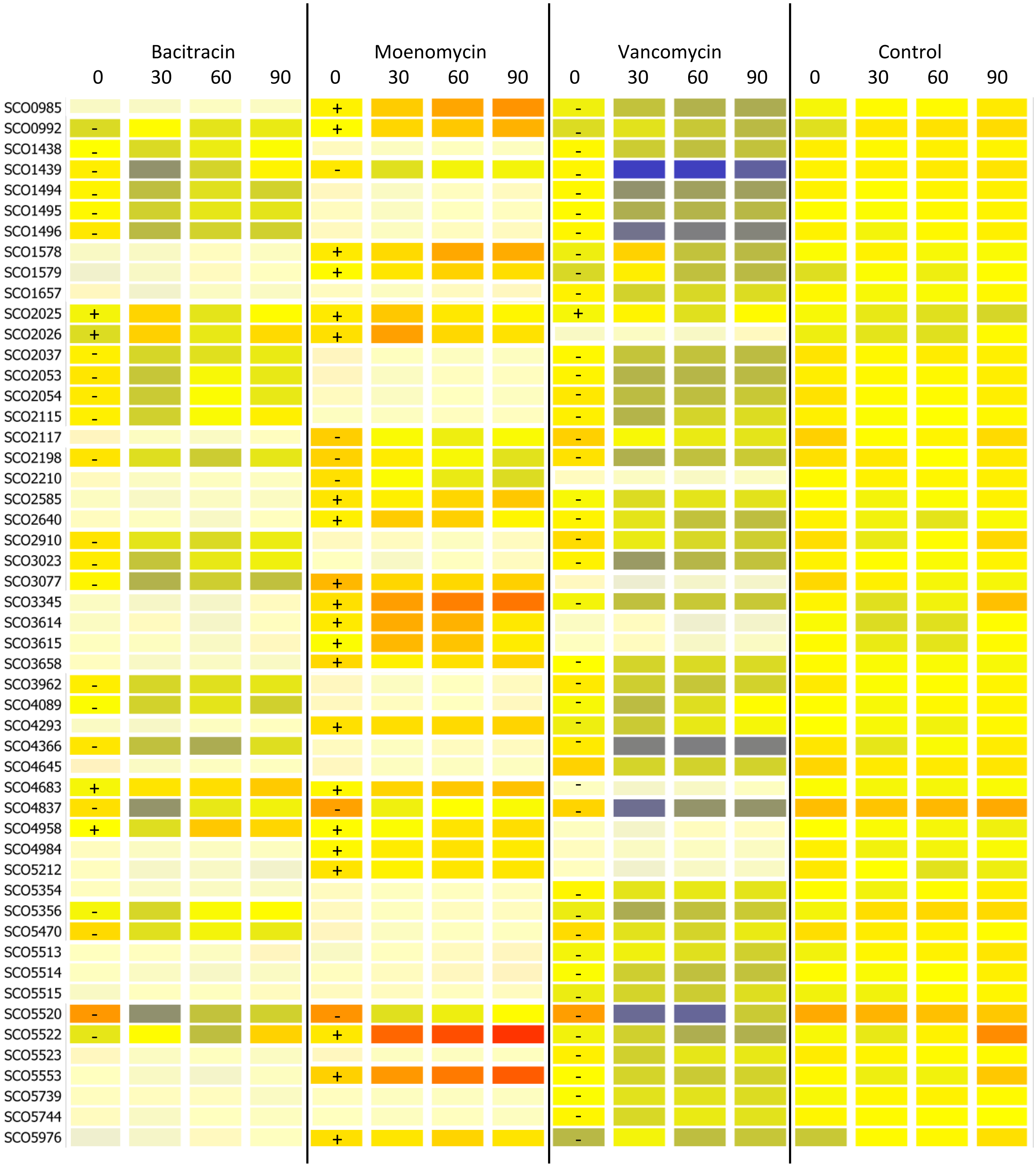

d) Calcium-dependent antibiotic CDA

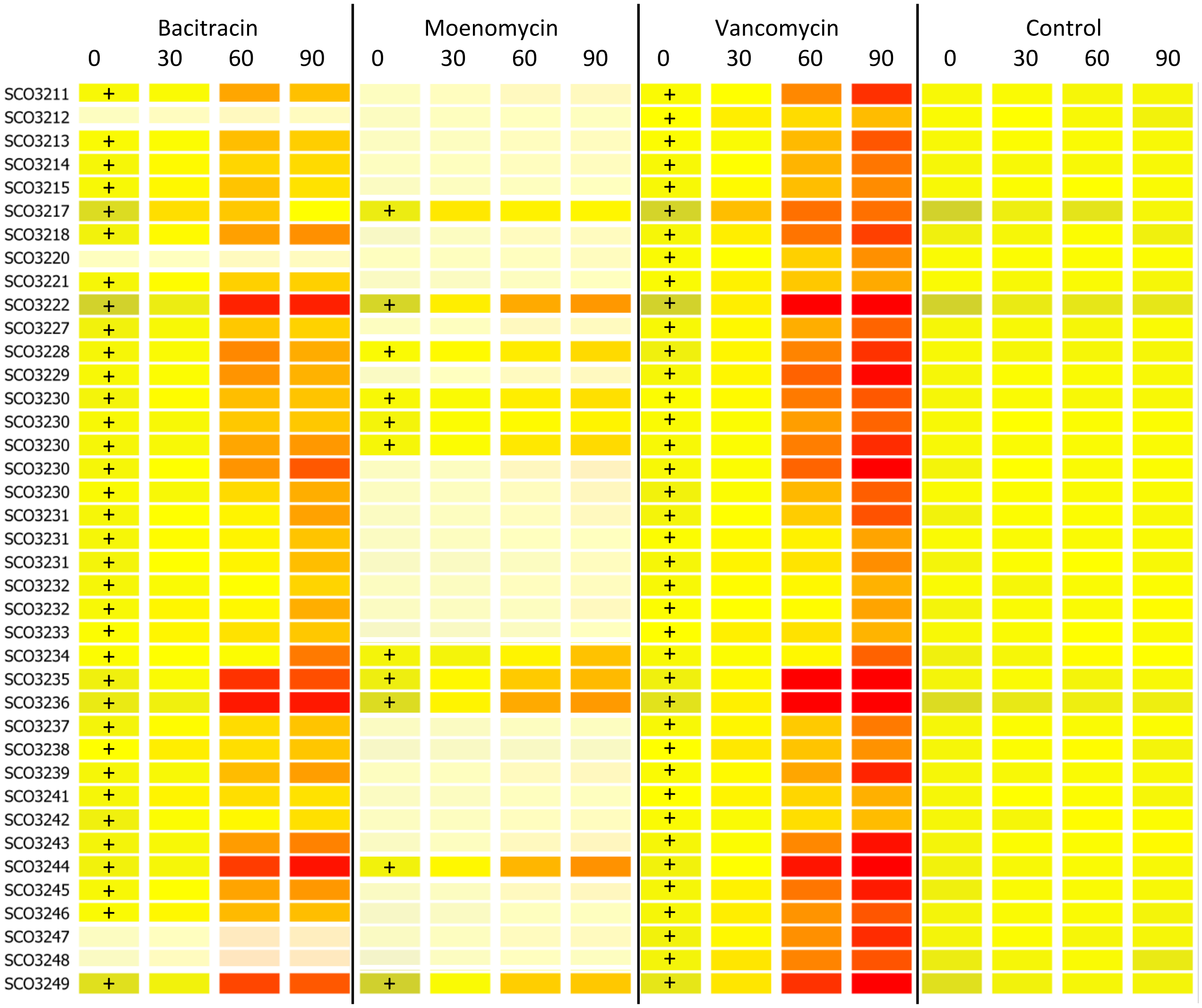

e) Cell wall hydrolases

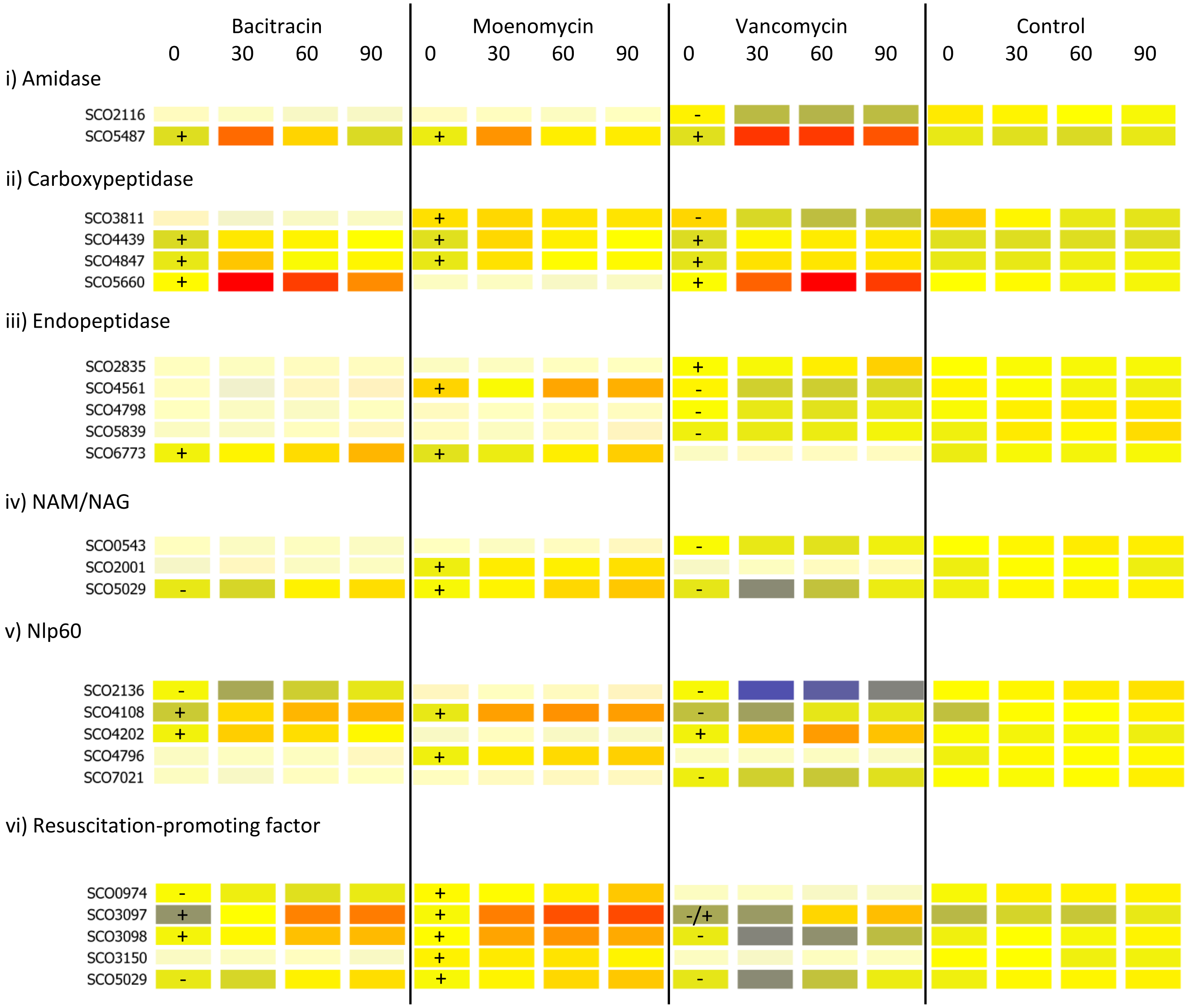

f) Conservons

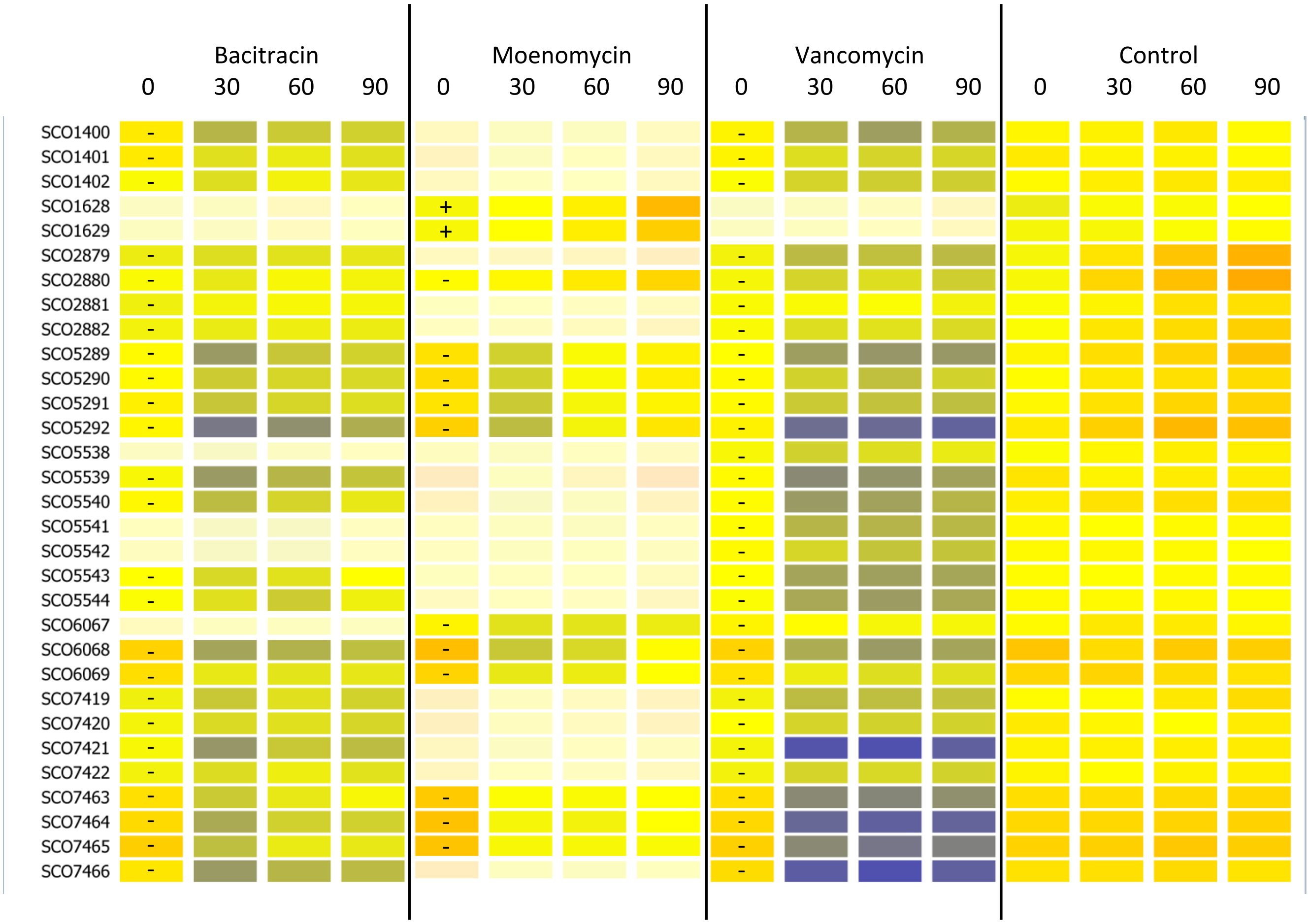

g) Ectoine biosynthesis

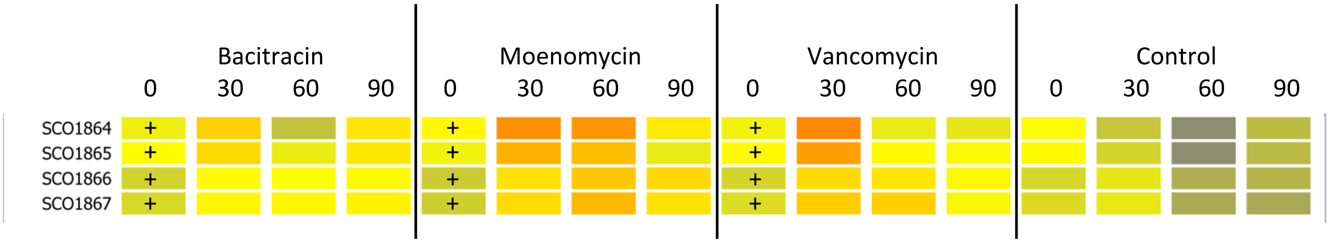

h) Gas vesicle 1

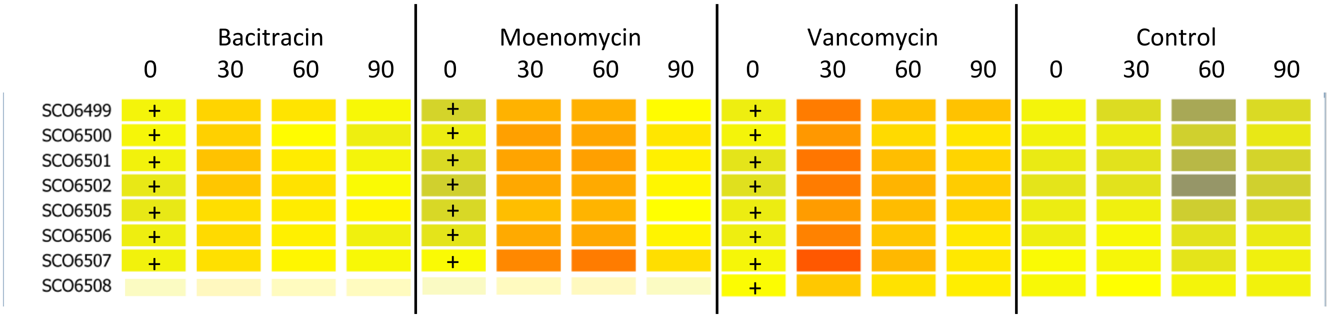

i) Red antibiotic biosynthetic cluster

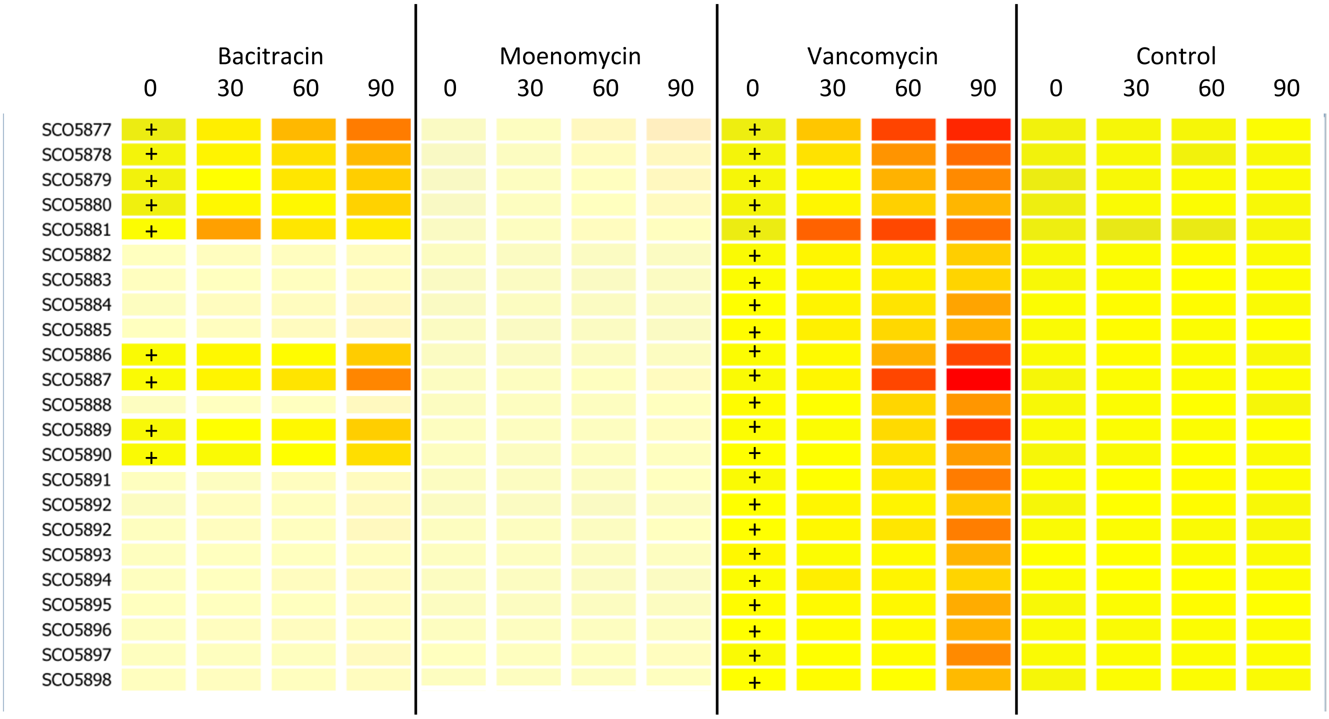

j) Response regulators

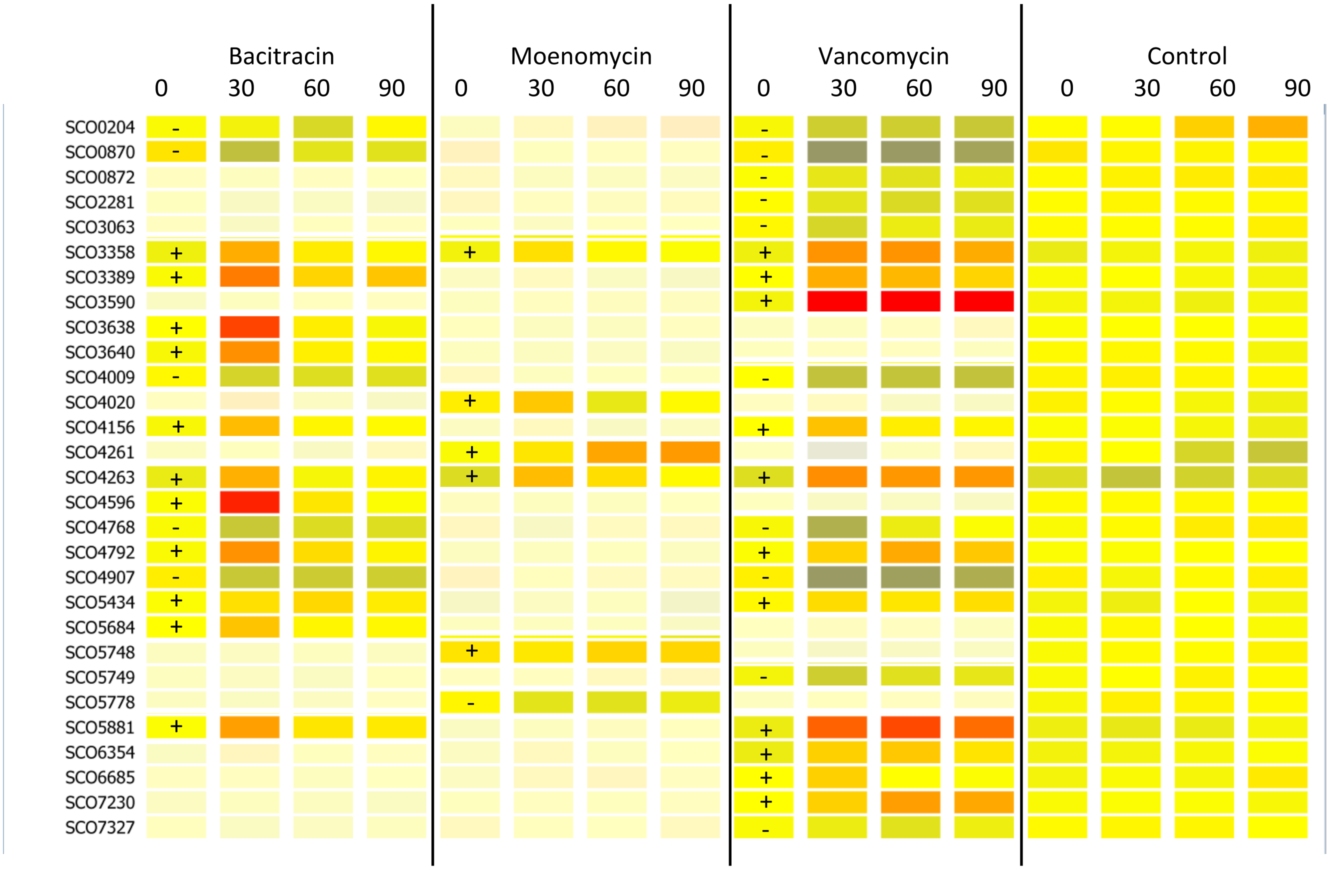

k) Sensor kinases

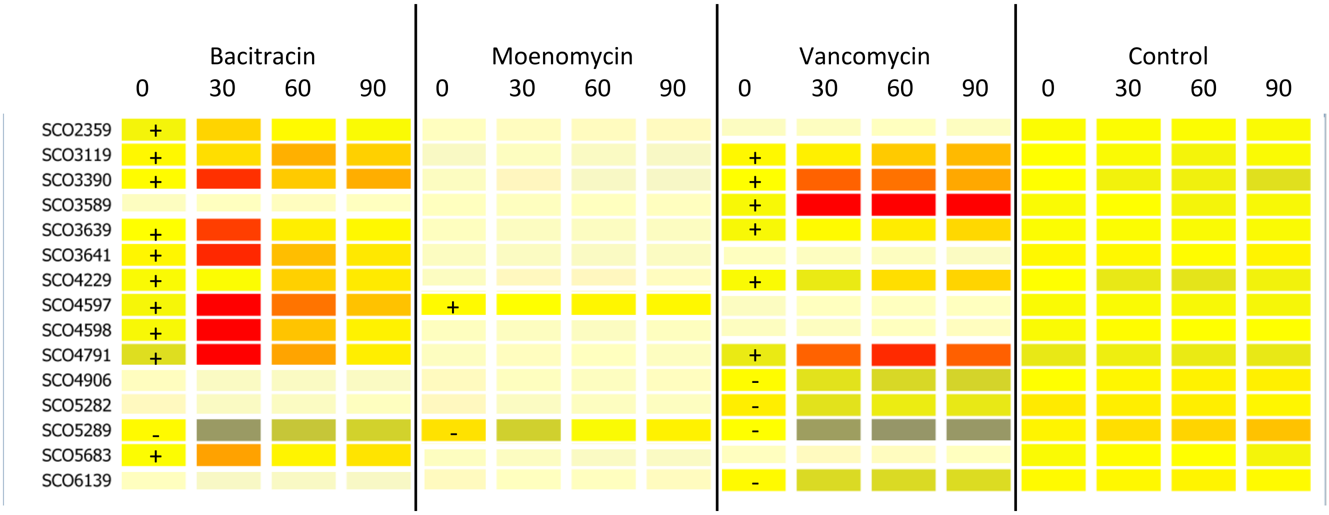

l) Sigma factors

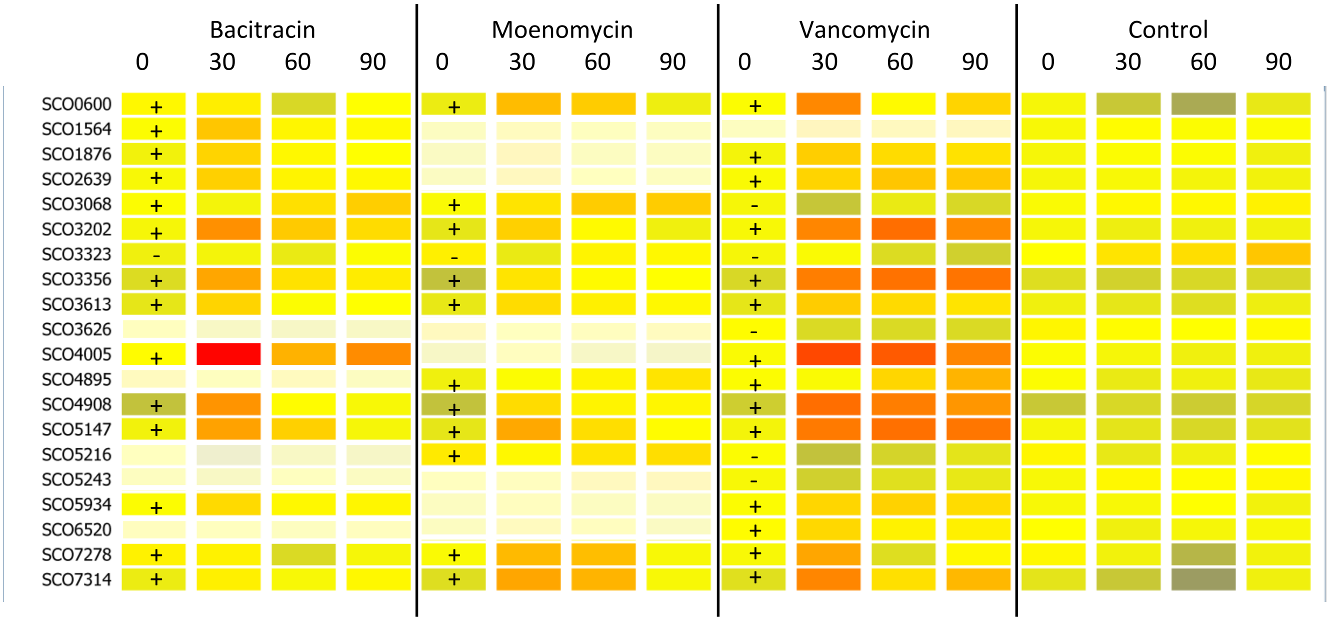

m) SigR regulon

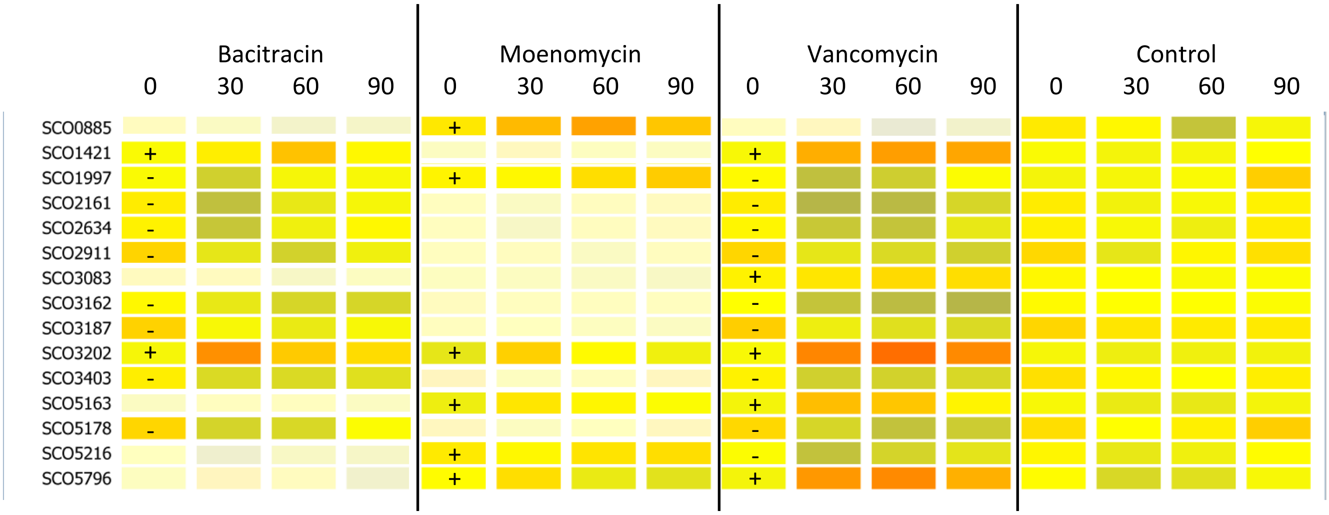

n) Sortase substrate proteins

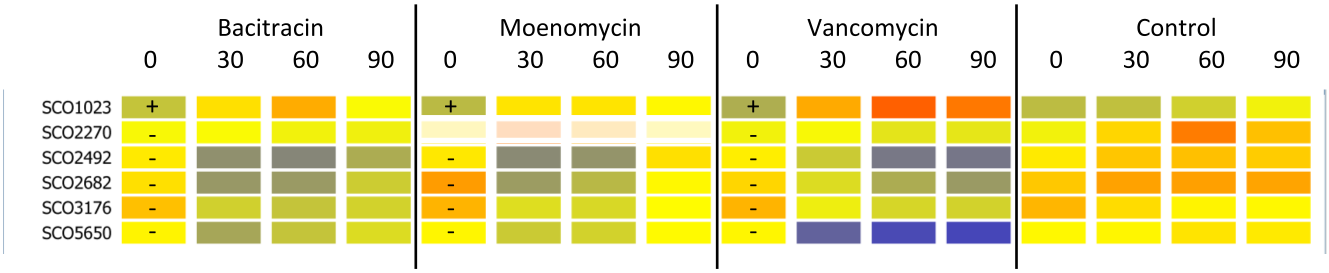

o) SsgA like

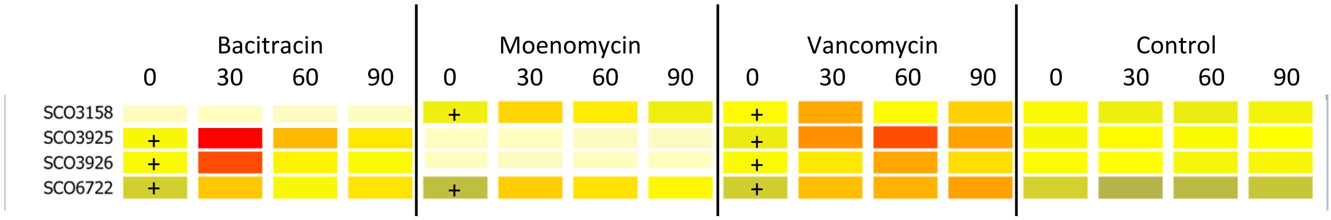

p) Zur regulon

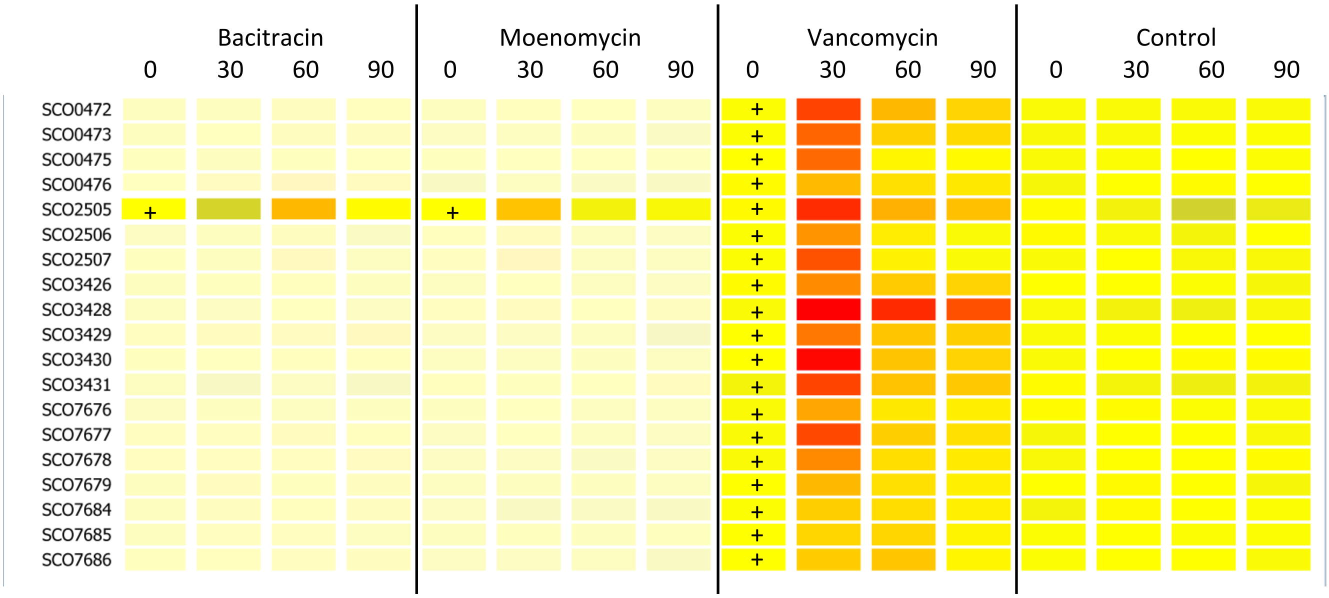

Supplement: Additional file 14 — Heatmaps summarising the expression of genes significantly differently expressed in response to drug treatment, grouped according to related function. [file 1471-2164-12-226-S14.PDF]
